# Use of Health Savings Accounts Among US Adults Enrolled in High-Deductible Health Plans

**DOI:** 10.1001/jamanetworkopen.2020.11014

**Published:** 2020-07-17

**Authors:** Jeffrey T. Kullgren, Elizabeth Q. Cliff, Christopher Krenz, Brady T. West, Helen Levy, Mark Fendrick, Angela Fagerlin

**Affiliations:** 1 Veterans Affairs (VA) Center for Clinical Management Research, VA Ann Arbor Healthcare System, Ann Arbor, Michigan; 2 Department of Internal Medicine, University of Michigan Medical School, Ann Arbor; 3 Department of Health Management and Policy, University of Michigan School of Public Health, Ann Arbor; 4 University of Michigan Institute for Healthcare Policy and Innovation, Ann Arbor; 5 Center for Bioethics and Social Sciences in Medicine, University of Michigan Medical School, Ann Arbor; 6 Department of Health Policy and Administration, University of Illinois at Chicago; 7 Institute for Social Research, University of Michigan, Ann Arbor; 8 Salt Lake City VA Center for Informatics Decision Enhancement and Surveillance, Salt Lake City, Utah; 9 Department of Population Health Sciences, University of Utah, Salt Lake City

## Abstract

**Importance:**

Health savings accounts (HSAs) can be used by enrollees in high-deductible health plans (HDHPs) to save for health care expenses before taxes. Expansion of and encouraging contributions to HSAs have been centerpieces of recent federal legislation. Little is known about how US residents who may be eligible for HSAs are using them to save for health care.

**Objective:**

To determine which patients who may be eligible for an HSA do not have one and what decisions patients with HSAs make about contributing to them.

**Design, Setting, and Participants:**

This cross-sectional national survey assessed an online survey panel representative of the US adult population. Adults aged 18 to 64 years and enrolled in an HDHP for at least 12 months were eligible to participate. Data were collected from August 26 to September 19, 2016, and analyzed from November 1, 2019, to April 30, 2020.

**Main Outcomes and Measures:**

Prevalence of not having an HSA or not making HSA contributions in the last 12 months and reasons for not making the HSA contributions.

**Results:**

Based on data from 1637 individuals (American Association of Public Opinion Research response rate 4, 54.8%), half (50.6% [95% CI, 47.7%-53.6%]) of US adults in HDHPs were female, and most were aged 36 to 51 (35.7% [95% CI, 32.8%-38.6%]) or 52 to 64 (36.8% [95% CI, 34.1%-39.5%]) years. Approximately 1 in 3 (32.5% [95% CI, 29.8%-35.3%]) did not have an HSA. Those who obtained their health insurance through an exchange were more likely to lack an HSA (70.3% [95% CI, 61.9%-78.6%]) than those who worked for an employer that offered only 1 health insurance plan (36.5% [95% CI, 30.9%-42.1%]; *P* < .001). More than half of individuals with an HSA (55.0% [95% CI, 51.1%-58.8%]) had not contributed money into it in the last 12 months. Among HDHP enrollees with an HSA, those with at least a master’s degree (46.1% [95% CI, 38.3%-53.9%]; *P* = .02) or a high level of health insurance literacy (47.3% [95% CI, 40.7%-54.0%]; *P* = .03) were less likely to have made no HAS contributions. Common reasons for not contributing to an HSA included not considering it (36.8% [95% CI, 30.8%-42.8%]) and being unable to afford saving for health care (31.9% [95% CI, 26.2%-37.6%]).

**Conclusions and Relevance:**

These findings suggest that many US adults enrolled in an HDHP lack an HSA, and few with an HSA saved for health care in the last year. Targeted interventions should be explored by employers, health plans, and health systems to encourage HSA uptake and contributions among individuals who could benefit from their use.

## Key Points

**Question:**

How are US adults who are enrolled in a high-deductible health plan using health savings accounts to save for health care?

**Findings:**

In this national survey of 1637 respondents, approximately 1 in 3 adults enrolled in a high-deductible health plan did not have a health savings account. Among those with a health savings account, most had not contributed money into it in the last year, and less education and health insurance literacy were associated with not having made contributions.

**Meaning:**

These findings suggest that few US adults enrolled in high-deductible health plans are using health savings accounts to save for health care, and targeted interventions could enhance uptake of and contributions to health savings accounts.

## Teaser

This survey study assesses which patients who may be eligible for a health savings account do not have one and what decisions patients with these accounts make about contributing to them in a representative sample of US adults.

## Introduction

Health savings accounts (HSAs) can be used by US residents who are enrolled in a high-deductible health plan (HDHP) to accrue tax-free savings for health care expenses, with the policy goals of lessening patients’ cost burdens and encouraging health care choices that are based on value. The proportion of privately insured adults who are enrolled in an HDHP increased from 25.3% in 2010 to 40.0% in 2016.^1^ The tax-advantaged saving opportunities of HSAs could help facilitate use of needed health care services^2^ among this growing number of HDHP enrollees, especially those who pay federal income tax, as well as preempt the cost-related access barriers and financial burdens that many experience,^3,4,5,6^ particularly those with lower income levels^6,7^ or a chronic condition.^8,9,10^

Beyond the potential benefits for patients who are enrolled in an HDHP,^1,11^ HSAs have been a frequent focus of recent federal health reform proposals. For example, in the 116th US Congress, at least 23 separate bills have been introduced to expand eligibility for HSAs to more than just HDHP enrollees, increase HSA contribution limits, or broaden the list of services that can be paid for with funds from an HSA.^12^ Expanding use of HSAs was also part of the president’s federal budgets for fiscal years 2020^13^ and 2021.^14^

Previous analyses have suggested that many individuals with HSAs could be saving more for health care,^15^ and that larger HSA balances are associated with more use of health care services.^2^ Much less is known about which individuals who may be eligible to have an HSA lack one^16^ or how individuals with HSAs make choices about their health care savings.^15^ This information is critical to the success of policy reforms to expand uptake of and contributions to HSAs. In addition, data on the use of HSAs could help employers, health plans, and health systems develop targeted interventions to encourage use of HSAs as 1 strategy to help more US residents navigate the rising costs of health care.

## Methods

### Data

This study was deemed exempt from review by the University of Michigan Medical School institutional review board owing to the use of deidentified survey data. This survey study followed the American Association for Public Opinion Research (AAPOR) reporting guideline.

From August 26 to September 19, 2016, we surveyed 1637 participants in the GfK KnowledgePanel who had been enrolled in an HDHP (defined as a private insurance plan with a deductible of at least $1300 for an individual or $2600 for a family) for at least 12 months, spoke English, and were aged 18 to 64 years. The GfK KnowledgePanel is an online survey panel with approximately 55 000 US adults constructed and weighted to be representative of the US population. The GfK achieves this representativeness through address-based sampling and provision of computers and internet access to participants who lack them. People with common chronic conditions (defined as anxiety, depression, asthma, chronic obstructive pulmonary disease, heart disease, type 1 or 2 diabetes, or hypertension) were oversampled because they often face greater financial burdens or forgo needed health care services when facing high cost sharing.^3,6,8,9,10^ Participants provided oral or written consent to GfK to participate in the KnowledgePanel and to receive periodic survey invitations from GfK. Additional details about the GfK KnowledgePanel are available in eMethods in the Supplement. The American Association for Public Opinion Research survey response rate 4, defined as the number of completed plus partially completed surveys out of the number of individuals who were known to be eligible plus an estimate of the proportion of individuals with unknown eligibility who were eligible,^17^ was 54.8% (eFigure in the Supplement).

### Survey Measures

Respondents were asked if they had an HSA, defined in the National Health Interview Survey as “a special account or fund that can be used to pay for medical expenses” that are “sometimes referred to as health savings accounts (HSAs), health reimbursement accounts (HRAs), personal care accounts, personal medical funds, or choice funds, and are different from flexible spending accounts.”^1^^(p10)^ Respondents were also asked if they saved for health care expenses in the last 12 months and, if so, which savings vehicles they used and how much they saved. Respondents who had not saved for health care in the last 12 months (in an HSA or any other savings vehicle) were asked to choose reasons why they had not saved from a list and could also write in reasons. Other survey questions asked about health status and source of insurance coverage, as well as factors that could potentially mediate health care savings, such as levels of consumer engagement,^18^ financial literacy,^19^ and health insurance literacy.^20^ To optimize participant understanding and internal validity, all new survey measures were refined through iterative cognitive interviews with 17 US adults in HDHPs with varying age, sex, sources of health insurance, geography, health literacy, and health care experience. New measures were then finalized after additional testing among 100 Amazon Mechanical Turk participants^21^ and 60 GfK KnowledgePanel participants. Survey responses were supplemented with demographic data collected by GfK, including participants’ self-report of their race and ethnicity. The full survey instrument is available in the eAppendix in the Supplement.

### Statistical Analysis

To compute nationally representative estimates, we used survey weights generated by GfK that accounted for the sampling design and survey nonresponse. Weights were calibrated to distributions of demographic variables from the March 2016 Current Population Survey and the 2015 National Health Interview Survey. To assess potential nonresponse bias, characteristics of survey respondents and nonrespondents were compared (eTable 1 in the Supplement), and the weighted sample was compared with weighted characteristics of nonelderly National Health Interview Survey participants enrolled in an HDHP (eTable 2 in the Supplement).

Weighted multivariable logistic regression was used to estimate the association between not having an HSA and demographic characteristics, health status, source of insurance, having a chronic condition, level of consumer engagement, level of health insurance literacy, and level of financial literacy. Among the subpopulation of individuals with an HSA, weighted multivariable logistic regression was used to estimate the association of not having made any HSA contributions in the last 12 months and these same independent variables. All independent variables were operationalized as mutually exclusive categories. Scores from scales used to measure potential mediators of saving (ie, consumer engagement, financial literacy, and health insurance literacy) were grouped into tertiles.^22,23,24^ Using the estimated coefficients from each regression model, we report marginal estimates of the adjusted prevalence of each outcome as a function of each independent variable.^25^

Analyses were conducted from November 1, 2019, to April 30, 2020. Stata, version 15.1 (StataCorp LLC), was used for all analyses. Variance estimates were computed using Taylor series linearization to reflect the variability of the survey weights. Two-tailed *P* values were obtained from logistic regression models, and *P* < .05 indicated significance.

## Results

### Population Characteristics

Based on weighted data from 1637 survey respondents, we estimate that half (50.6% [95% CI, 47.7%-53.6%]) of US adults in HDHPs were female, and most were aged 36 to 51 (35.7% [95% CI, 32.8%-38.6%]) or 52 to 64 (36.8% [95% CI, 34.1%-39.5%]) years. Most were employed (83.8% [95% CI, 81.6%-86.0%]) and received their health insurance through an employer (84.6% [95% CI, 82.6%-86.5%]). Nearly half (42.4% [95% CI, 39.6%-45.3%]) had a chronic condition. Additional characteristics of the study population have been previously published^22,23,24^ and are available in eTable 3 in the Supplement. Compared with survey nonrespondents, survey respondents were older (aged 52-64 years, 48.6% [955 CI, 46.2%-51.0%] vs 32.9% [95% CI, 31.6%-34.2%]), more likely to be men (53.9% [95% CI, 51.5%-56.3%] vs 43.5% [42.1%-44.9%]) and white (81.6% [95% CI, 79.7%-83.5%] vs 69.0% [95% CI, 67.7%-70.3%]), and had higher levels of education (bachelor’s degree, 34.1% [95% CI, 31.9%-36.4%] vs 28.8% [95% CI, 27.6%-30.1%]) and income (≥400% of the federal poverty line, 53.7% [95% CI, 51.3%-56.1%] vs 46.1% [95% CI, 44.7%-47.5%]) (eTable 1 in the Supplement).

### Prevalence of Lacking an HSA Overall and Among Subgroups

Among US adults with an HDHP, an estimated 32.5% (95% CI, 29.8%-35.3%) did not have an HSA, 58.4% (95% CI, 55.4%-61.3%) had an HSA, and 9.1% (95% CI, 7.5%-11.0%) did not know whether they had an HSA or did not complete the survey question about having an HSA. Compared with HDHP enrollees who worked for an employer that offered only 1 plan (36.5% [95% CI, 30.9%-42.1%]), those who worked for an employer that offered multiple plans were less likely to lack an HSA (21.5% [95% CI, 18.3%-24.7%]; *P* < .001), and those who obtained coverage through an exchange were more likely to lack an HSA (70.3% [95% CI, 61.9%-78.6%]; *P* < .001) (Table 1). Individuals with a master’s degree (22.1% [95% CI, 17.0%-27.3%]) were significantly less likely than those with a high school education or less (40.0% [95% CI, 32.6%-47.4%]; *P* < .001) to lack an HSA. There were no statistically significant differences in the adjusted prevalence of not having an HSA for individuals with lower incomes compared with those with higher incomes or for those with vs without chronic conditions. All estimated model parameters are shown in eTable 4 in the Supplement.

**Table 1.** Marginal Estimates of the Adjusted Prevalence of Reporting No HSA Among US Adults in HDHPs

Characteristic	Sample size^a^	No. of respondents^b^	Weighted % (95% CI)^c^	*P* value^d^
Educational level				
High school or less	295	130	40.0 (32.6-47.4)	NA^**e**^
Some college	404	164	33.7 (28.9-38.4)	.15
Bachelor’s degree	534	176	31.7 (27.2-36.2)	.07
Master’s degree or higher	314	73	22.1 (17.0-27.3)	<.001
Race/ethnicity				
White	1269	446	32.5 (29.7-35.2)	NA^**e**^
Black	83	31	30.7 (18.3-43.2)	.79
Hispanic	102	34	32.4 (23.3-41.5)	.96
Other	93	32	35.6 (24.9-46.3)	.57
Source of health insurance				
Employer without plan choice	377	141	36.5 (30.9-42.1)	NA^**e**^
Employer with choice of plans	877	188	21.5 (18.3-24.7)	<.001
Insurance exchange^f^	187	144	70.3 (61.9-78.6)	<.001
Other source	106	70	62.9 (52.6-73.2)	<.001
Health status				
Excellent	192	57	25.3 (18.4-32.2)	NA^**e**^
Very good	641	201	28.8 (25.1-32.5)	.36
Good	561	217	39.0 (34.3-43.8)	.004
Fair	130	57	38.6 (29.1-48.2)	.03
Poor	23	11	32.2 (14.9-49.6)	.45
Chronic condition				
Yes	757	287	31.5 (27.8-35.3)	.48
No	790	256	33.5 (29.8-37.3)	NA^**e**^
Level of health insurance literacy^g^				
Lowest tertile	513	182	31.8 (27.3-36.4)	NA^**e**^
Middle tertile	517	197	35.5 (31.1-40.0)	.26
Highest tertile	517	164	30.4 (25.8-35.0)	.67
Level of financial literacy^h^				
Lowest tertile	302	108	31.2 (25.4-37.0)	1 [Reference]
Middle tertile	400	156	34.4 (29.4-39.3)	.41
Highest tertile	845	279	32.4 (28.6-36.1)	.75

Abbreviations: HDHPs, high-deductible health plans; HSA, health savings account; NA, not applicable.

^a^ Overall sample size for this analysis (n = 1547) and for each subgroup is based on the number of survey respondents with nonmissing values for independent variables in the multivariable logistic regression model.

^b^ An HSA was defined as in the National Health Interview Survey as “a special account or fund that can be used to pay for medical expenses” that are “sometimes referred to as health savings accounts (HSAs), health reimbursement accounts (HRAs), personal care accounts, personal medical funds, or choice funds, and are different from flexible spending accounts.”^1^^(p10)^

^c^ Indicates weighted prevalence of not having an HSA adjusted for all variables listed in Table 1 in addition to age, sex, income, race/ethnicity, region, and level of consumer engagement^18^ using a logistic regression model to assess the likelihood of not having an HSA. Estimates of coefficients for each of the predictor variables in the model can be found in eTable 4 in the Supplement.

^d^ Calculated using logistic regression models.

^e^ Indicates reference category.

^f^ Respondents answered they had “health insurance that you bought through a state or federal individual marketplace/exchange.”

^g^ Indicates tertiles of participants’ scores for the Health Insurance Literacy Measure.^20^

^h^ Tertiles of participants’ sum of scores for 3 measures of financial literacy developed by Lusardi and Mitchell.^19^

### Prevalence of Making No HSA Contributions Overall and Among Subgroups

**Figure 1.**
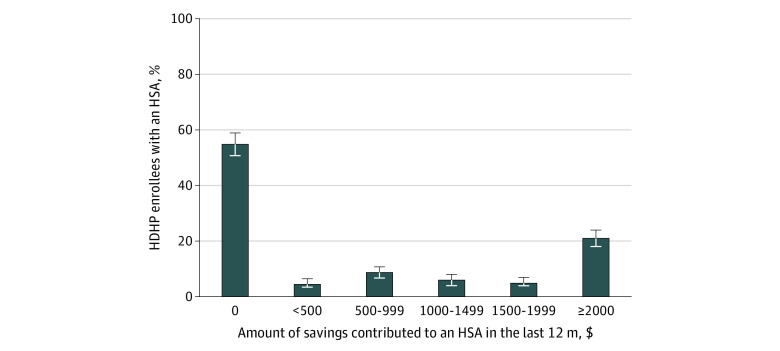
Health Care Savings in the Last Year Among US Adults in High-Deductible Health Plans (HDHPs) Who Had a Health Savings Account (HSA) Includes 929 respondents with proportions weighted for US population. Error bars indicate 95% CIs.

Among HDHP enrollees with an HSA, more than half (55.0% [95% CI, 51.1%-58.8%]) had not contributed any money into it in the last 12 months (Figure 1). Compared with HDHP enrollees with an HSA who had no more than a high school education (62.6% [95% CI, 52.4%-72.8%]), those who had a bachelor’s degree (49.5% [95% CI, 43.2%-55.7%]; *P* = .04) or at least a master’s degree (46.1% [95% CI, 38.3%-53.9%]; *P* = .02) were less likely to have made no HSA contributions in the last 12 months (Table 2). Individuals with an HSA who had a high level of health insurance literacy (47.3% [95% CI, 40.7%-54.0%]) were significantly less likely to have made no HSA contributions in the last 12 months than those with a low level of health insurance literacy (58.6% [95% CI, 51.5%-65.6%]; *P* = .03). Compared with HDHP enrollees with an HSA who had obtained their coverage from an employer that offered only 1 insurance plan (61.0% [95% CI, 53.2%-68.7%]), those who had obtained their coverage from an exchange (30.9% [95% CI, 6.9%-54.9%]) were less likely to have made no HSA contributions in the last 12 months (*P* = .03). There were no statistically significant differences in the adjusted prevalence of having made no HSA contributions in the last 12 months by income level or the presence of a chronic condition. All estimated model parameters are shown in eTable 5 in the Supplement.

**Table 2.** Marginal Estimates of the Adjusted Prevalence of Making No HSA Contributions in the Last Year Among US Adults in HDHPs Who Had an HSA

Characteristic	Sample size	No. of respondents	Weighted % (95% CI)^a^	*P* value^b^
Educational level				
High school or less	134	88	62.6 (52.4-72.8)	NA^**c**^
Some college	198	116	59.1 (51.3-66.9)	.59
Bachelor’s degree	316	147	49.5 (43.2-55.7)	.04
Master’s degree or higher	216	94	46.1 (38.3-53.9)	.02
Race/ethnicity				
White	716	360	52.3 (48.2-56.4)	NA^**c**^
Black	42	25	66.6 (51.2-81.9)	.10
Hispanic	56	29	44.9 (30.7-59.0)	.33
Other	50	31	66.7 (49.9-83.5)	.13
Source of health insurance				
Employer without plan choice	187	108	61.0 (53.2-68.7)	NA^**c**^
Employer with choice of plans	629	320	52.2 (47.8-56.6)	.06
Insurance exchange^d^	21	6	30.9 (6.9-54.9)	.03
Other source	27	11	52.6 (33.5-71.6)	.41
Health status				
Excellent	117	64	57.1 (46.7-67.5)	NA^**c**^
Very good	390	192	54.4 (48.6-64.0)	.65
Good	286	147	50.7 (44.1-57.4)	.33
Fair	59	31	51.6 (35.8-67.4)	.58
Poor	12	11	75.1 (35.8-115.5)	.46
Chronic condition				
Yes	409	218	53.2 (46.8-59.5)	.81
No	455	227	54.2 (49.2-59.3)	NA^**c**^
Level of health insurance literacy^e^				
Lowest tertile	266	155	58.6 (51.5-65.6)	NA^**c**^
Middle tertile	276	146	56.9 (50.5-63.3)	.73
Highest tertile	322	144	47.3 (40.7-54.0)	.03
Level of financial literacy^f^				
Lowest tertile	140	89	58.1 (47.8-68.3)	NA^**c**^
Middle tertile	206	119	57.4 (49.6-65.3)	.92
Highest tertile	518	237	51.1 (46.0-56.2)	.26

Abbreviations: HDHPs, high-deductible health plans; HSA, health savings account; NA, not applicable.

^a^ Based on marginal effects from a logistic regression model in which the dependent variable was $0 in savings in an HSA in the past 12 months, if a respondent reported having an HSA and responded to questions about savings. Savings level of $0 was defined as either reporting not saving any money in the last 12 months for health care or not saving any money for health care through their HSA. Sample was anyone who reported having an HSA and had nonmissing values for covariates in model (n = 864). Survey weights were based on the full sample of respondents with nonmissing covariates (n = 1564). Prevalences are adjusted for age, sex, income, region, and level of consumer engagement.^18^ Estimates of coefficients for each of the predictor variables in the model can be found in eTable 5 in the Supplement.

^b^ Calculated using logistic regression models.

^c^ Indicates reference category.

^d^ Respondents answered they had “health insurance that you bought through a state or federal individual marketplace/exchange.”

^e^ Indicates tertiles of participants’ scores for the Health Insurance Literacy Measure.^20^

^f^ Indicates tertiles of participants’ sum of scores for 3 measures of financial literacy developed by Lusardi and Mitchell.^19^

### Reasons for Not Contributing to HSAs

**Figure 2.**
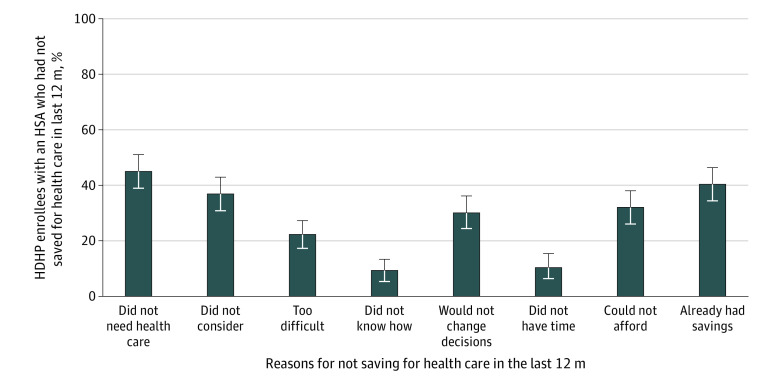
Reasons for Not Saving for Health Care Among US Adults in High-Deductible Health Plans (HDHPs) Who Had a Health Savings Account (HSA) Includes 366 respondents with proportions weighted for US population. Error bars indicate 95% CIs.

The most common reasons for not saving in the last 12 months among people with an HSA were that they did not perceive a need for health care services (44.9% [95% CI, 38.7%-51.1%]) or that they already had sufficient savings to cover health care expenses (40.2% [95% CI, 34.2%-46.3%]) (Figure 2). Other common reasons for not contributing to an HSA included not considering it (36.8% [95% CI, 30.8%-42.8%]) and being unable to afford saving for health care (31.9% [95% CI, 26.2%-37.6%]).

### Savings Among HDHP Enrollees Who Had an HSA

**Figure 3.**
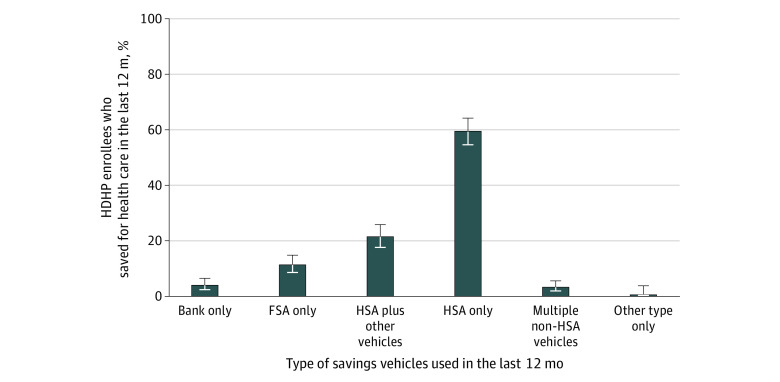
Savings Vehicles Used by US Adults in High-Deductible Health Plans (HDHPs) Who Had a Health Savings Account (HSA) and Saved for Heath Care Includes 548 respondents with proportions weighted for US population. Error bars indicate 95% CIs; FSA, flexible spending account.

Of those with an HSA who did contribute money into their account in the last 12 months, the most frequent level of contribution was $2000 or more (46.7% [95% CI, 41.2%-52.2%]). For most individuals who contributed to their HSA (59.4% [95% CI, 54.5%-64.2%]), the HSA was the only place they saved for health care (Figure 3). Another 21.4% (95% CI, 17.6%-25.8%) of those who put money into their HSA used another vehicle to save for health care (eg, a flexible spending account or bank account) in addition to their HSA.

## Discussion

In this survey study, few US adults who were enrolled in HDHPs were using HSAs to save for health care expenses, despite the cost-related barriers to access and financial burdens experienced by the growing number of HDHP enrollees.^3,4,5,6,7,10^ Previous analyses have examined levels of HSA contributions and balances^15^ and their relation to the use of health care services^2^ among people who have HSAs. This study is among the first, to our knowledge, to examine how US adults who may be eligible for HSAs are using these accounts to save for future health care expenses and identifies opportunities for new interventions that employers, health plans, and health systems could implement to encourage use of HSAs among individuals who are eligible to have them and could benefit from their use.

Approximately 1 in 3 US adults who were enrolled in an HDHP reported not having an HSA. Some HDHP enrollees were more likely than others to lack an HSA, particularly individuals who obtained their insurance through an exchange. The relatively high rate of exchange plans lacking an HSA may be because, although the mean deductible for a federal health insurance Marketplace individual plan was $5316 during the 2020 open enrollment period, just 7% of Marketplace plans chosen for 2020 were qualified for linkage to an HSA.^26^ This low rate of HSA eligibility could be owing to some Marketplace HDHPs covering services before the deductible in ways that are not currently allowed in HSA-eligible plans.^27^ Consequently, a policy option for increasing uptake of HSAs in Marketplace plans would be to allow more flexibility in what types of HDHPs qualify for linkage to an HSA.

Nevertheless, our results also suggest that legislative efforts to expand eligibility for HSAs may not have their full intended effects without concomitant efforts to support eligible individuals to take advantage of their eligibility and facilitate their acquisition of an HSA. In the case of HDHPs obtained through an exchange, exchanges could better highlight the benefits of HSAs to encourage uptake of HSA-eligible plans at the time of enrollment. Alternatively, policy reforms could require that HDHPs offered on an exchange be eligible for and linked to an HSA. After enrollment in an HDHP, employers, health plans, and health systems could target messaging to HDHP enrollees to encourage acquisition of an HSA as a strategy to help manage the high cost-sharing of these plans. Although we collected data on reasons for not contributing to an HSA among those who had one, we were not able to collect data on why individuals without an HSA had not acquired one. This is an important area for future research, which would provide valuable information on whether these interventions, or other strategies, can help facilitate uptake of HSAs among individuals who are eligible to have them.

Most individuals who reported having an HSA had not contributed any savings into their account in the previous 12 months. Although some of these individuals may have had a positive balance in their account from previous contributions,^15^ already having sufficient savings was only cited by 40% of individuals as a reason for not having saved for health care in the last 12 months. This result suggests that current legislative efforts to increase the HSA contribution limit would likely have little effect on health care savings for the vast majority of people with HSAs and that other factors may be driving consumers’ decisions about HSA savings. Indeed, additional common reasons for not having saved for health care included not having considered saving and being unable to afford saving. To prompt consideration of contributing to an HSA, health plans and health systems could periodically target educational messaging to HDHP enrollees who have an HSA. Among individuals who have an employer-sponsored HDHP with an HSA but have not contributed to it, employers could facilitate more HSA savings through interventions that have been successful at increasing retirement savings in workplaces, such as default contributions to HSAs, employer matching of employee HSA contributions, or committing future wage increases to savings.^28^

Approximately 1 in 5 individuals with an HSA who saved for health care in the last 12 months reported saving in a non-HSA vehicle along with their HSA. For individuals who have not reached their annual HSA contribution limit, this may be financially inferior to the triple tax advantage of saving in an HSA (ie, HSA contributions are not taxed, HSA earnings are not taxed, and qualified HSA withdrawals are not taxed). Another 20% of people with an HSA reported saving for health care only in a non-HSA vehicle and could similarly be missing opportunities to reduce their tax burdens. Together, these patterns suggest that employers and health plans have potentially important roles to play in helping individuals with HSAs better understand these accounts and make financially advantageous decisions about their saving for health care.

Although people with lower incomes or chronic conditions can be especially vulnerable to cost-related access barriers when enrolled in an HDHP,^6,7,8,9,10^ individuals in these subgroups were not less likely than others to lack an HSA. Similarly, among individuals who had an HSA, those with lower incomes or chronic conditions were not any less likely than others to have made no contributions to their HSA in the last year. Efforts by employers, health plans, and health systems to encourage uptake and use of HSAs should particularly focus on these subgroups of HDHP enrollees who could potentially benefit from the use of HSAs as a strategy to lessen their known cost-related access barriers and financial burdens.

### Limitations

These data are self-reported and thus subject to recall bias. To limit such bias, existing survey measures were used where possible, and new measures were developed through extensive cognitive interviews.^22^ To classify people as having an HSA, an existing National Health Interview Survey measure was used that relied on individuals’ knowledge, because it was not feasible to collect details about their accounts. Although this approach is consistent with many other studies,^1,29,30^ it means that we were unable to confirm that participants’ HSAs were Internal Revenue Service–qualified HSAs. If someone reported having multiple savings vehicles, including an HSA, we assumed that all reported savings for health care went into their HSA, which would bias reported HSA savings upward. To check the sensitivity of our results to this assumption, we analyzed levels of savings among those who only saved in an HSA and found that this did not change our main conclusions. We did not inquire about reasons for not enrolling in an HSA or, among people with an HSA, balances or expenditures for their HSA, and thus were unable to examine these issues.

## Conclusions

The results of this survey study suggest that few US adults enrolled in HDHPs are using HSAs to save for their health care expenses. As enrollment in HDHPs and policy interest in expanding use of HSAs both continue to grow, employers, health plans, and health systems should explore targeted interventions to encourage HSA uptake and contributions among individuals whose use of an HSA could potentially improve the affordability of needed health care services.

## Notes

Supplement. **eMethods.** Additional Details **eFigure.** Response Rate Flowchart **eAppendix.** Survey Instrument **eTable 1.** Characteristics of Survey Respondents and Nonrespondents **eTable 2.** Characteristics of Survey Sample and Nonelderly 2015 NHIS Participants in HDHPs **eTable 3.** Characteristics of Americans in High-Deductible Health Plans (n = 1637) **eTable 4.** Coefficients From Logit Regression Model to Predict Not Having an HSA (n = 1547) **eTable 5.** Coefficients From Logit Regression Model to Predict Not Saving in an HSA in the Last 12 Months (n = 864)
