# Supplementary material for: Use of Health Savings Accounts Among US Adults Enrolled in High-Deductible Health Plans
Source: JAMA Netw Open. 2020 Jul 17;3(7):e2011014. doi: 10.1001/jamanetworkopen.2020.11014 (PMC7368175; doi:10.1001/jamanetworkopen.2020.11014)
Supplement: Supplement. — eMethods. Additional Details eFigure. Response Rate Flowchart eAppendix. Survey Instrument eTable 1. Characteristics of Survey Respondents and Nonrespondents eTable 2. Characteristics of Survey Sample and Nonelderly 2015 NHIS Participants in HDHPs eTable 3. Characteristics of Americans in High-Deductible Health Plans (n = 1637) eTable 4. Coefficients From Logit Regression Model to Predict Not Having an HSA (n = 1547) eTable 5. Coefficients From Logit Regression Model to Predict Not Saving in an HSA in the Last 12 Months (n = 864) [file jamanetwopen-3-e2011014-s001.pdf]

Supplementary Online Content

Kullgren JT, Cliff EQ, Krenz C, et al. Use of health savings accounts among US adults enrolled in high-deductible health plans. *JAMA Netw Open*. 2020;3(7):e2011014. doi:10.1001/jamanetworkopen.2020.11014

eMethods. Additional Details

eFigure. Response Rate Flowchart

eAppendix. Survey Instrument

eTable 1. Characteristics of Survey Respondents and Nonrespondents

eTable 2. Characteristics of Survey Sample and Nonelderly 2015 NHIS Participants in HDHPs

eTable 3. Characteristics of Americans in High-Deductible Health Plans (n = 1637)

eTable 4. Coefficients From Logit Regression Model to Predict Not Having an HSA (n = 1547)

eTable 5. Coefficients From Logit Regression Model to Predict Not Saving in an HSA in the Last 12 Months (n = 864)

This supplementary material has been provided by the authors to give readers additional information about their work.

©2020 Kullgren JT et al. *JAMA Network Open*.

eMethods. Additional Details

KnowledgePanel Methodology

KnowledgePanel (KP) is the largest U.S. online panel that relies on probability-based sampling techniques for recruitment; hence, the largest national sampling frame from which fully representative samples can be generated to produce statistically valid inferences for study populations. This panel provides samples with the highest level of representativeness available in online research for measurement of public opinions, attitudes, and behaviors. The panel was first developed in 1999 by Knowledge Networks, a GfK company. Panel members are randomly selected so that survey results can properly represent the US population with a measurable level of accuracy, features that are not obtainable from nonprobability panels.

The recruitment process for KP was originally based exclusively on a national Random Digit Dialing (RDD) sampling methodology. In order to improve the representation of the panel, GfK migrated to using an Address Based Sampling (ABS) methodology via the Delivery Sequence File (DSF) of the USPS for recruiting panel members in 2009. This probability-based sampling methodology improves population coverage, and provides a more effective sampling infrastructure for recruitment of hard-to-reach individuals, such as young adults and those from various minority groups. Under the ABS recruitment, households without Internet connection are provided with a web-enabled device and free Internet service.

After initially accepting the invitation to join the panel, participants are asked to complete a short demographic survey (the initial Core Profile Survey), answers to which allow efficient panel sampling and weighting for future surveys. Completion of the Core Profile Survey allows participants to become active panel members.

Survey Sampling from KnowledgePanel

Once KP members are recruited and profiled by taking the Core Profile Survey, they become eligible for selection for client surveys. Typically, survey samples are based on an equal probability of selection method (EPSEM) sample from the panel for general population surveys. Customized stratified random sampling based on profile data can also be carried out as required by the study design to reduce screening costs for rare subgroups.

For this particular survey, a nationally representative sample of U.S. adults (18 to 64) with private health insurance was selected. The sample was also selected so that half the respondents had previously reported one of the following chronic conditions on the Core Profile Survey, and half had not:

- Anxiety Disorder
- Asthma/Bronchitis/COPD
- Depression
- Heart Attack
- Heart Disease
- High Blood Pressure
- Type 1 or Type 2 Diabetes

Survey Administration

Once assigned to a survey, KP members receive a notification email letting them know there is a new survey available for them to take. This email notification contains a link that sends them to the survey questionnaire. No login name or password is required. The field period depends on the client's needs and can range anywhere from a few hours to several weeks. After three days, automatic email reminders are sent to all non-responding panel members in the sample. If email reminders do not generate a sufficient response, an automated telephone reminder call can be initiated. The usual protocol is to wait at least three to four days after the email reminder before calling. To assist panel members with their survey taking, each individual has a personalized "home page" that lists all the surveys that were assigned to that member and have yet to be completed.

GfK also operates an ongoing modest incentive program to encourage participation and create member loyalty. Members can enter special raffles or can be entered into special sweepstakes with both cash rewards and other prizes to be won. The

typical survey commitment for panel members is one survey per week or four per month with duration of 10 to 15 minutes per survey. In the case of longer surveys, an additional incentive is typically provided.

Sample Weighting

Significant resources and infrastructure are devoted to the recruitment process for KP so that active panel members can properly represent the U.S. adult population. This representation is not only achieved with respect to a broad set of geodemographic indicators, but also harder-to-reach adults such as those without Internet access or who speak only Spanish. Consequently, the distribution of KP participants closely mirrors the distribution of the U.S. population, barring occasional differences that may emerge for certain subgroups due to differential attrition.

For selection of general population samples from KP a patented methodology has been developed that ensures all samples behave as EPSEM. Briefly, this methodology starts by weighting the pool of active members to the geodemographic benchmarks secured from the latest March supplement of the Current Population Survey (CPS) along several dimensions. Using the resulting weights as measure of size, in the next step a PPS (probability proportional to size) procedure is used to select study specific samples. It is the application of this PPS methodology with the imposed size measures that produces fully self-weighting samples from KP, for which each sample member can carry a design weight of unity. Moreover, in instances where a study design requires any form of oversampling of certain subgroups, such departures from an EPSEM design are accounted for by adjusting the design weights in reference to the CPS benchmarks for the population of interest.

The geodemographic benchmarks used to weight the active panel members for computation of size measures include:

- Gender (Male/Female)
- Age (18–29, 30–44, 45–59, and 60+)
- Race/Hispanic ethnicity (White/Non-Hispanic, Black/Non-Hispanic, Other/Non-Hispanic, 2+ Races/Non-Hispanic, Hispanic)
- Education (Less than High School, High School, Some College, Bachelor and beyond)
- Census Region (Northeast, Midwest, South, West)
- Household income (under $10k, $10K to <$25k, $25K to <$50k, $50K to <$75k, $75K to <$100k, $100K to <$150k, and $150K+)
- Home ownership status (Own, Rent/Other)
- Metropolitan Area (Yes, No)

Study-Specific Post-Stratification Weights

Once the study sample has been selected and the survey administered, and all the survey data are edited and made final, design weights are adjusted to account for any differential nonresponse that may have resulted during the field period. Depending on the specific target population for a given study, geodemographic distributions for the corresponding population are obtained from the CPS, the American Community Survey (ACS), or in certain instances from the weighted KP profile data. For this purpose an iterative proportional fitting (raking) procedure is used to produce the final weights. In the final step, calculated weights are examined to identify and, if necessary, trim outliers at the extreme upper and lower tails of the weight distribution. The resulting weights are then scaled to aggregate to the total sample size of all eligible respondents.

For this study, the following benchmark distributions from the most recent data from the CPS and NHIS were used for the raking adjustment:

<u>CPS</u>

- Age (18-24; 25-34; 35-44,45-54,55-64) by Gender (Male, Female)
- Race (White/Non-Hispanic, Black/Non-Hispanic, Other/Non-Hispanic, Hispanic, 2+ Races/Non-Hispanic)
- Census Region (Northeast, Midwest, South, West) by Metropolitan Status (Metro/Non-Metro)
- Education (Less than High School, High School, Some College, Bachelor or higher)
- Household Income (under $25K, $25-$49,999, $50K-$74,999, $75K)

<u>NHIS</u>

©2020 Kullgren JT et al. *JAMA Network Open*.

- Chronic Conditions (Asthma, Chronic Bronchitis, Chronic Obstructive Pulmonary Disease, Diabetes, Coronary Heart Disease, or High Blood Pressure)

eFigure. Response Rate Flowchart[a]

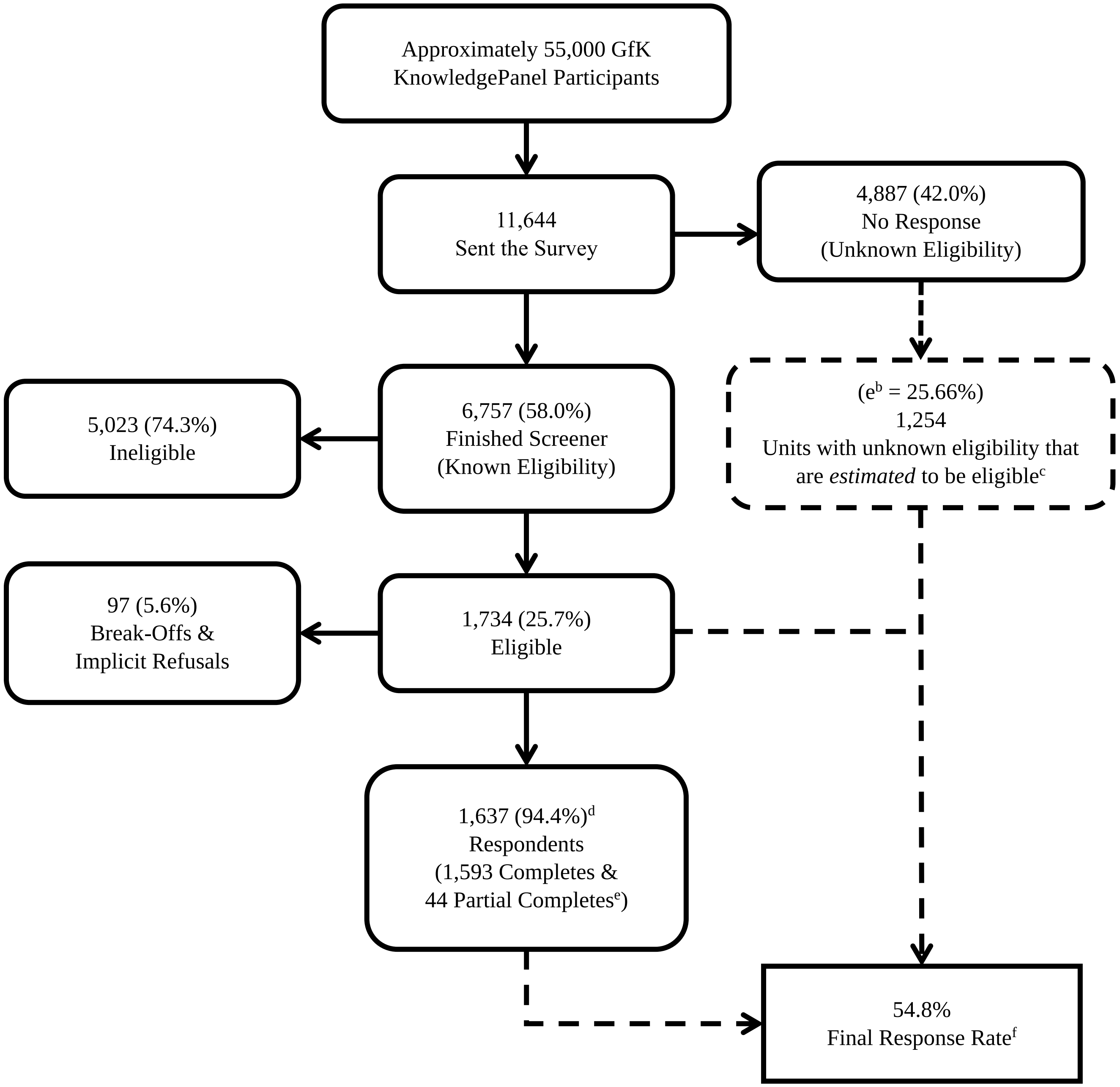

[a]Standard Definitions: Final Dispositions of Case Codes and Outcome Rates for Surveys. 9th edition. [Internet]. The American Association f or Public Opinion Research; 2016. Available from: http://www.aapor.org/AAPOR_Main/media/publications/Standard-Definitions20169theditionfinal.pdf

[b]"e is the estimated proportion of cases of unknown eligibility that are eligible. […] This estimate is based on the proportion of eligible units among all units in the sample for which a definitive determination of status was obtained (a conservative estimate)." e = Eligible / Finished Screener (i.e. participants with known eligibility), or 1,734 / 6,757 = 25.66%.

©2020 Kullgren JT et al. *JAMA Network Open*.

[c]Units with unknown eligibility that are estimated to be eligible = e * No Response (Unknown Eligibility), or
1,254 = 25.66% * 4,887.
[d]AAPOR Cooperation Rate 4.
[e]"Partial Completes" includes individuals who broke-off before completing the survey but after answering the questions on the consumer behaviors that were the main measures in our survey.
[f]AAPOR Response Rate 4.

©2020 Kullgren JT et al. *JAMA Network Open*.

eAppendix. Survey Instrument

IV. SCREENER

SCRIPTER: Show inserts in regular font unless otherwise specified
Prompt once for all the Questions.

BASE: All respondents

QINS1 [S]
Below is a list of different kinds of health insurance. Which of the following is your primary source of insurance coverage?

1. Health insurance through your or someone else's employer or union
2. Medicare, a government plan that pays health care bills for people aged 65 or older and for some disabled people
3. Medicaid or any other state medical assistance plan for those with lower incomes
4. Health insurance that you bought through a state or federal individual marketplace/exchange
5. Veteran's Affairs (VA), Department of Defense, or other military programs
6. Health insurance from some other source
7. I do not have any health care insurance/coverage

[if QINS1=2 or 3 or 5 or 7 or refused, terminate and insert standard close].

BASE: Total Qualified Respondents

QINS2 [S]
Do you have supplemental insurance/coverage in addition to what you listed above?

1. Yes
2. No
3. I don't know

[if QINS2=1 or 3 or Refused, terminate and insert standard close].

BASE: Total Qualified respondents

Q19 [M]
Have YOU been diagnosed by a doctor or other qualified medical professional with any of the following medical conditions?

1. Anxiety disorder
2. Asthma, chronic bronchitis, or COPD
3. Depression
4. Diabetes
5. Heart attack
6. Heart disease
7. High blood pressure
8. None of these **[S]**

IF Q19 =1-7 DOV Chronic= 1; IF Q19 =8 DOV Chronic = 2.

Scripter: 50% Respondents should be assigned to Dov_chronic =1 (Have chronic conditions(s), and the remaining 50% respondents should be assigned to Dov Chronic =2 (does not have chronic disease)

BASE: Total Qualified Respondents

S1_Scope [S]

©2020 Kullgren JT et al. *JAMA Network Open*.

Are you the only one who is covered under your health insurance plan or is there at least one other person?

1. Just me
2. Me and at least one other person

BASE: Total Qualified respondents

S2_Deductible [s]
A deductible is the amount of money you have to pay before your health insurance will pay anything for your health care.

Is the annual deductible for medical care for your plan less than **[if S1_Scope=1:** $1,300 **/if S1_Scope=2:** $2,600**]** or **[if S1_Scope=1:** $1,300 **/if S1_Scope=2:** $2,600**]** or more?

When answering this question do not think about any separate deductibles you might have for prescription drugs, hospitalization, or out-of-network care.

1. Less than **[if S1_Scop =1:** $1,300 **/if S1_Scope=2:** $2,600**]**
2. **[if S1_Scope=1:** $1,300 **/if S1_Scope=2:** $2,600**]** or more
3. Do not know

[if SELECTED S2_Deductible=2 continue or else terminate and insert standard close].

BASE: Total Qualified respondents

S3_Duration [s]
Have you been enrolled in a health insurance plan with a deductible of **[if S1_Scope=1:** $1,300 **/if S1_Scope=2:** $2,600**]** or more for at least the past 12 months?

1. Yes
2. No

[if SELECTED S2_ Duration=1 continue or else terminate and insert standard close].

BASE: Total Qualified respondents

[Display]

Thank you for your interest in our survey on health care decisions. This survey will take about 20 minutes to complete. Filling out the survey is your choice. You can skip any questions that you don't want to answer. In order to protect your privacy, we will not connect your name or email address to your survey. Clicking "Next" below means that you agree to take the survey.

If you have any questions about this study, please do not hesitate to contact our Research Coordinator, Chris Krenz, at krenzc@med.umich.edu.

Jeff Kullgren, MS, MD, MPH
University of Michigan
jkullgre@med.umich.edu

BASE: Total Qualified respondents

First we have some questions about your health insurance plan.

BASE: Ask if QINS1=1(" Health insurance through your or someone else's employer or union")

©2020 Kullgren JT et al. *JAMA Network Open*.

MultiplePlans [s]
Does the employer who provided your health insurance plan offer more than one plan to its employees?
1. Yes
2. No
3. Don't know

BASE: Total Qualified respondents

Hsa [s]
With your plan, is there a special account or fund that can be used to pay for medical expenses? The accounts are sometimes referred to as Health Savings Accounts (HSAs), Health Reimbursement Accounts (HRAs), Personal Care accounts, Personal Medical funds, or Choice funds, and are different from Flexible Spending Accounts.

1. Yes
2. No
3. Don't know

A. Shopping Behaviors

BASE: Total Qualified respondents

Shopping Behaviors [Grid, S across]

Next we would like to ask you some questions about shopping and money.

Please tell us **how much you disagree or agree** with the following statements.

Down:
SHOP1. In whatever I buy, I shop a lot for specials.
SHOP2. I find myself checking prices in the grocery store even for small items.
SHOP3. I like to use coupons as much as possible.
SHOP4. I generally shop a lot to compare products.
SHOP5. I divide my shopping between 2 or 3 stores to get the best deal.

Across:
1. Strongly Disagree
2. Disagree
3. Neutral
4. Agree
5. Strongly Agree

B. Ace

BASE: Total Qualified respondents

Ace [Grid, S across]

Now we'd like to ask you some questions about how you think about your health.

Please tell us **how much you disagree or agree** with the following statements.

Down:
ACE1. I spend a lot of time learning about health.
ACE2. Even when life is stressful, I know I can continue to do the things that keep me healthy.
ACE3. I feel comfortable talking to my doctor about my health.

©2020 Kullgren JT et al. *JAMA Network Open*.

ACE4. When I work to improve my health, I succeed.
ACE5. I have brought my own information about my health to show my doctor.
ACE6. When choosing a new doctor, I look for information online.
ACE7. I can stick with plans to exercise and eat a healthy diet.
ACE8. I compare doctors using official ratings about how well their patients are doing.
ACE9. I have lots of experience using the health care system.
ACE10. When choosing a new doctor, I look for official ratings based on patient health.
ACE11. Different doctors give different advice; it's up to me to choose what's right for me.
ACE12. I handle my health well.

Across:
1. Strongly Disagree
2. Disagree
3. Neither Agree nor Disagree
4. Agree
5. Strongly Agree

C. Health Literacy

BASE: Total Qualified respondents

HL_Forms [Grid, S across]

How confident are you filling out medical forms by yourself?

Across:

1. Extremely
2. Quite a bit
3. Somewhat
4. A little bit
5. Not at all

D. Use

BASE: Total Qualified respondents

[Display]
The next questions are about your use of health care services in the past 12 months. As you answer these questions, **think only about your own individual health care** and not health care that was for someone else (e.g., a child, parent, or friend).

BASE: Total Qualified respondents

Use_Num[Banked Grid, S across]

During the past 12 months…

Down:

Use_NumOutpatient How many times have you seen a doctor or other health care professional about your own health at a doctor's office, a clinic, or some other place? Do not include times you were hospitalized overnight, visits to hospital emergency rooms, home visits, dental visits, or telephone calls. Your best estimate is fine.

Use_NumInpatient How many different times did you stay in any hospital overnight or longer? Do not include any overnight stays in the emergency room. Your best estimate is fine.

©2020 Kullgren JT et al. *JAMA Network Open*.

Use_NumED How many times have you gone to a hospital emergency room about your own health? Please include emergency room visits that resulted in a hospital admission. Your best estimate is fine.

Across:

1. None
2. 1
3. 2
4. 3
5. 4 or more
6. Don’t know

E. Unmet

BASE: Total Qualified respondents

Unmet [Grid, S across]

During the past 12 months…

Down:

Unmet_Delay
Have you delayed seeking medical care because of worry about the cost?

Unmet_Foregone
Was there any time when you needed medical care, but did not get it because you couldn't afford it?

Unmet_Medbills
Did you have problems paying or were unable to pay any medical bills? Include bills for doctors, dentists, hospitals, therapists, medication, equipment, nursing home or home care.

Across

1. Yes
2. No

F. Behaviors Intro

The next set of questions is about things that some people do and some people do not do when thinking about and getting health care services (e.g., a doctor visit, procedure, test, or medication). For these questions, please tell us whether you have done these while thinking about and getting health care services **in the past 12 months.** There are no right answers and we just want to understand your experiences so that we can help other patients make decisions about health care services.
For this next set of questions, **think only about your own individual health care** and not health care that was for someone else (e.g., a child, parent, or friend).

BASE: Total Qualified respondents

B_Engage [S]
In the past 12 months, did you **put aside money** to pay for any health care services (e.g., a doctor visit, procedure, test, or medication) before you needed them (e.g., in a Health Savings Account or Flexible Spending Account, bank account, or somewhere else)?

1. Yes

©2020 Kullgren JT et al. *JAMA Network Open*.

2. No

BASE: if B_Engage=1 ("yes")
Scripter: please insert mouseover for BY_HSA and BY_FSA

BY_Account [S]
In the past 12 months, did you put aside money to pay for health care services, before you needed them, in any of the following? If needed, you can scroll over 'Health Savings Account (HSA)' and 'Flexible Spending Account (FSA)' for more detail.

Down:
BY_HSA. **[mouseover]**Health Savings Account (HSA)
BY_FSA. **[mouseover]**Flexible Spending Account (FSA)
BY_Bank. Personal bank account
BY_Other. Somewhere else

Across:
1. Yes
2. No

Scripter:
mouseover text for BY_HSA:
Health Savings Account (HSA): An HSA is a tax-advantaged account where money can be put aside to pay for qualifying medical expenses. In order to quality for an HSA, you must have a high-deductible health plan (HDHP). Either individuals or employers can contribute pre-tax dollars to the account, and money withdrawn for qualified medical expenses is never taxed. Unlike a Flexible Spending Account, contributions roll over from year to year.

mouseover text for BY_FSA:
Flexible Spending Account (FSA): An FSA is a tax-advantaged account where money can be put aside to pay for qualifying medical expenses. An FSA is established by an employer and is funded through pre-tax payroll deductions or employer contributions. Unlike a Health Savings Account, funds in an FSA must be used by the end of the year (or the end of the next grace period) or they will be lost.

BASE: if BY_Other=1 ("Yes")

BY_Other_Specify [O]
Where else did you **put aside money** in the past 12 months to pay for health care services before you needed them?

BASE: Total Qualified respondents

BY_Savings [S]
How much money in total did you put aside in the past 12 months to pay for health care services before you needed them?

1. Less than $500
2. $500 to $999
3. $1,000 to $1,499
4. $1,500 to $1,999
5. $2,000 or more

BASE: if B_Engage=1 ("yes")

BY_Effects/Facilitators [Grid,S across]
Did **putting aside money** in the past 12 months to pay for health care services before you needed them result in any of the following things for you?

©2020 Kullgren JT et al. *JAMA Network Open*.

Down:
BYE_GetCare. Getting a health care service you felt you needed.
BYE_PutOff. Deciding to put off getting a health care service until you could afford it.
BYE_Forego. Deciding a health care service wasn't worth the cost.
BYE_LessCost. Paying less for a health care service.
BYE_LessDebt. Having less debt from health care bills.
BYE_OtherCosts. Freeing up money you used to pay for other (non-health care) things.

Across:
1. Yes
2. No

BASE: if B_Engage=1 ("yes") show on same page with by_Effects

BYE_Other[O]
Please describe anything else that resulted from **putting aside money** in the past 12 months to pay for health care services before you needed them (if nothing else resulted, just leave this blank):

BASE: if B_Engage=1 ("yes")

[Grid,S across]
How much did each of the following things help you **put aside money** in the past 12 months to pay for health care services before you needed them?

Down:
BYF_Web. A website
BYF_App. A smart phone app
BYF_Friend. A family member, friend, or acquaintance
BYF_Employer. An employer who contributed to an account for health care spending
BYF_HealthPlan. Your health insurance plan
BYF_Staff. Someone at your doctor's office
BYF_Broker. An expert in money (e.g., financial adviser, broker, etc.)

Across:
1. Not at all
2. A little

3. A lot

BASE: if B_Engage=1 ("yes") show on same page with by_facilitators

BYF_Other [O]
Please describe anything else that helped you **put aside money** in the past 12 months to pay for health care services before you needed them (if nothing else helped you, just leave this blank):

BASE: if BYF_Web=2 or 3 ("A little" or "A lot")
Scripter : show BYF_Web_Specify - BYF_Broker_Specify on same page

BYF_Web_Specify [O]
What website(s) helped you?

BASE: if BYF_App=2 or 3 ("A little" or "A lot")

BYF_App_Specify [O]
What smart phone app(s) helped you?

©2020 Kullgren JT et al. *JAMA Network Open*.

BASE: if BYF_Staff=2 or 3 (“A little” or “A lot”)

BYF_Staff_Specify [O]
Who in your doctor’s office helped you?

BASE: if BYF_Broker =2 or 3 (“A little” or “A lot”)

BYF_Broker_Specify [O]
What type of expert in money helped you?

BASE: if B_Engage=2 (“No”)

BN_Barriers/Facilitators[Grid,S across]

Were any of the following things reasons why you did not **put aside money** in the past 12 months to pay for health care services before you needed them?

Down:
BNB_NoNeed. You didn’t need any health care services.
BNB_NotConsider. You didn’t think about it.
BNB_TooHard. You felt it would be too difficult.
BNB_NotKnowHow. You didn’t know how to.
BNB_NoImpact. It wouldn’t change your decisions.
BNB_NoTime. You didn’t have time to.
BNB_NoMoney. You couldn’t afford to.
BNB_AlreadySave. You already had enough savings to pay for health care services you might need.

Across:
1. Yes
2. No

BASE: if B_Engage=2 (“No”) show on same page as bn_barriers

BNB_Other [O]
Please describe any other reasons you did not **put aside money** to pay for health care services in the past 12 months before you needed them (if there were no other reasons, just leave this blank):

BASE: if B_Engage=2 (“No”)

[Grid,S across]

In the future, how much do you think each of the following things could help you **put aside money** to pay for health care services before you need them?

Down:
BNF_Web. A website
BNF_App. A smart phone app
BNF_Friend. A family member, friend, or acquaintance
BNF_Employer. An employer who contributes to an account for health care spending
BNF_HealthPlan. Your health insurance plan
BNF_Staff. Someone at your doctor’s office
BNF_Broker. An expert in money (e.g., financial adviser, broker, etc.)

Across:
1. Not at all

©2020 Kullgren JT et al. *JAMA Network Open*.

2. A little
3. A lot

BASE: if B_Engage=2 (“No”) show on same page with bn_facilitators

BNF_Other [O]
Please describe anything else that you think could help you **put aside money** to pay for health care services before you need them (if you can’t think of anything, just leave this blank):

G. PRICE_Comparison

[Display]
For this next set of questions, **think only about your own individual health care** and not health care that was for someone else (e.g., a child, parent, or friend).

BASE: Total Qualified respondents

P_Engage [S]
In the past 12 months, did you **compare prices** for any health care services (e.g., a doctor visit, procedure, test, or medication) at different places?

1. Yes
2. No

BASE: ask if P_Engage=1 (“yes”)

PY_Services[S]

For each of the following types of health care services, did you **compare prices** at different places in the past 12 months?

Down:
PY_Lab. A lab test (e.g., a blood or urine test)
PY_Image. An imaging or radiology test
PY_Meds. A prescription medicine
PY_Procedure. A procedure or surgery
PY_Outpatient. A visit to a doctor’s office, a clinic, or some other place
PY_Inpatient. A hospitalization
PY_Other. Something else

Across:
1. Yes
2. No

BASE: ask if PY_Other =1 (“Yes”)

PY_Other_Specify [S]
For what other type(s) of health care services did you compare prices at different places?

BASE: ask if P_Engage=1 (“yes”)

PY_Effects/Facilitators [S]
Did **comparing prices** for health care services at different places in the past 12 months result in any of the following things for you?

Down:

©2020 Kullgren JT et al. *JAMA Network Open*.

PYE_GetCare. Getting a health care service you felt you needed.
PYE_PutOff. Deciding to put off getting a health care service until you could afford it.
PYE_Forego. Deciding a health care service wasn't worth the cost.
PYE_LessCost. Paying less for a health care service.
PYE_LessDebt. Having less debt from health care bills.
PYE_OtherCosts. Freeing up money you used to pay for other (non-health care) things.

Across:
1. Yes
2. No

BASE: ask if P_Engage=1 ("yes") show on same screen as PY_effects

PYE_Other [O]

Please describe anything else that resulted from **comparing prices** for health care services at different places in the past 12 months (if nothing else resulted, just leave this blank)

BASE: ask if P_Engage=1 ("yes")

[Grid, S Across]
How much did each of the following things help you **compare prices** for health care services at different places in the past 12 months?

Down:
PYF_Web. A website
PYF_App. A smart phone app
PYF_Friend. A family member, friend, or acquaintance
PYF_HealthPlan. Your health insurance plan
PYF_Staff. Someone at your doctor's office
PYF_Broker. An expert in money (e.g., financial adviser, broker, etc.)

Across:
1. Not at all
2. A little
3. A lot

BASE: ask if P_Engage=1 ("yes") show on same screen as py_facilitators

PYF_Other[O]
Please describe anything else that helped you **compare prices** for health care services at different places in the past 12 months (if nothing else helped you, just leave this blank):

BASE: if PYF_Web=2 or 3 ("A little" or "A lot")
Scripter: show PYF_Web_Specify - PYF_Broker on same page

PYF_Web_Specify [O]
What website(s) helped you?

BASE: if PYF_App=2 or 3 ("A little" or "A lot")

PYF_App_Specify [O]
What smart phone app(s) helped you?

BASE: if PYF_Staff=2 or 3 ("A little" or "A lot")

PYF_Staff_Specify [O]
Who in your doctor's office helped you?

BASE: if PYF_Broker =2 or 3 ("A little" or "A lot")

PYF_Broker_Specify [O]
What type of expert in money helped you?

BASE: if P_Engage=2 ("no")

PN_Barriers/Facilitators[Grid,S across]

Were any of the following things reasons why you did not **compare prices** for health care services at different places in the past 12 months?

Down:
PNB_NoNeed. You didn't need any health care services.
PNB_NotConsider. You didn't think about it.
PNB_TooHard. You felt it would be too difficult.
PNB_NotKnowHow. You didn't know how to.
PNB_NoImpact. It wouldn't change your decisions.
PNB_NoTime. You didn't have time to.
PNB_NoVary. Prices for health care services don't vary much.

Across:
1. Yes
2. No

BASE: if P_Engage=2 ("no") show on same screen as pn_Barriers

PNB_Other [O]
Please describe any other reasons you did not **compare prices** for health care services at different places in the past 12 months (if there were no other reasons, just leave this blank):

BASE: if P_Engage=2 ("no")

[Grid,S across]

In the future, how much do you think each of the following things could help you **compare prices** for health care services at different places? Select one answer from each row in the grid.

Down:
PNF_Web. A website
PNF_App. A smart phone app
PNF_Friend. A family member, friend, or acquaintance
PNF_HealthPlan. Your health insurance plan
PNF_Staff. Someone at your doctor's office
PNF_Broker. An expert in money (e.g., financial adviser, broker, etc.)

Across:
1. Not at all
2. A little
3. A lot

©2020 Kullgren JT et al. *JAMA Network Open*.

BASE: if P_Engage=2 (“no”) show on same screen as pn_facilitator

PNF_Other [O]
Please describe anything else that you think could help you **compare prices** for health care services at different places (if you can’t think of anything, just leave this blank):

H. QUALITY_Comparison

[Display]
For this next set of questions, **think only about your own individual health care** and not health care that was for someone else (e.g., a child, parent, or friend).

BASE: Total Qualified respondents

Q_Engage [S]
In the past 12 months, did you **compare quality ratings** for any health care services (e.g., a doctor visit or procedure) at different places (e.g., different doctors or different hospitals)?

1. Yes
2. No

BASE: Q_Engage=1 (“yes”)

QY_Services [S]
For each of the following types of health care services, did you **compare quality ratings** at different places in the past 12 months?

Down:
QY_Procedure. A procedure or surgery
QY_Outpatient. A visit to a doctor’s office, a clinic, or some other place
QY_Inpatient. A hospitalization
QY_Other. Something else

Across:
1. Yes
2. No

BASE: QY_Other =1 (“Yes”) show on same screen as qy_services

QY_Other_Specify [O]
For what other type(s) of health care services did you compare quality ratings at different places?

BASE: ask if Q_Engage=1 (“yes”)

QY_Effects/Facilitators [S]
Did **comparing quality ratings** for health care services at different places in the past 12 months result in any of the following things for you?

Down:
QYE_GetCare. Getting a health care service you felt you needed.
QYE_PutOff. Deciding to put off getting a health care service until you could afford it.
QYE_Forego. Deciding a health care service wasn’t worth the cost.
QYE_LessCost. Paying less for a health care service.
QYE_LessDebt. Having less debt from health care bills.
QYE_OtherCosts. Freeing up money you used to pay for other (non-health care) things.

©2020 Kullgren JT et al. *JAMA Network Open*.

Across:
1. Yes
2. No

BASE: ask if Q_Engage=1 ("yes") show on same screen as qy_effect

QYE_Other [O]

Please describe anything else that resulted from **comparing quality ratings** for health care services at different places in the past 12 months (if nothing else resulted, just leave this blank)

BASE: ask if Q_Engage=1 ("yes")

[Grid, S Across]
How much did each of the following things help you **compare quality ratings** for health care services at different places in the past 12 months?

Down:
QYF_Web. A website
QYF_App. A smart phone app
QYF_Friend. A family member, friend, or acquaintance
QYF_HealthPlan. Your health insurance plan
QYF_Staff. Someone at your doctor's office

Across:
1. Not at all
2. A little
3. A lot

BASE: ask if Q_Engage=1("yes") show on same page as qy_facilitator
QYF_Other[O]
Please describe anything else that helped you **compare quality ratings** for health care services at different places in the past 12 months (if nothing else helped you, just leave this blank):

BASE: if QYF_Web=2 or 3 ("A little" or "A lot")

Scripter: show QYF_Web_Specify - QYF_Staff on same

QYF_Web_Specify [O]
What website(s) helped you?

BASE: if QYF_App=2 or 3 ("A little" or "A lot")

QYF_App_Specify [O]
What smart phone app(s) helped you?

BASE: if QYF_Staff=2 or 3 ("A little" or "A lot")

QYF_Staff_Specify [O]
Who in your doctor's office helped you?

BASE: if Q_Engage=2 ("No")

QN_Barriers/Facilitators[Grid,S across]

©2020 Kullgren JT et al. *JAMA Network Open*.

Were any of the following things reasons why you did not **compare quality ratings** for health care services at different places in the past 12 months?

Down:
QNB_NoNeed. You didn't need any health care services.
QNB_NotConsider. You didn't think about it.
QNB_TooHard. You felt it would be too difficult.
QNB_NotKnowHow. You didn't know how to.
QNB_NoImpact. It wouldn't change your decisions.
QNB_NoTime. You didn't have time to.
QNB_NoVary. Quality of health care services doesn't vary much.

Across:
1. Yes
2. No

BASE: if Q_Engage=1 ("No") show on same page as Qn_Barriers

QNB_Other [O]
Please describe any other reasons you did not **compare quality ratings** for health care services at different places in the past 12 months (if there were no other reasons, just leave this blank):

BASE: if Q_Engage=1 ("No")

[Grid,S across]

In the future, how much do you think each of the following things could help you **compare quality ratings** for health care services at different places?

Down:
QNF_Web. A website
QNF_App. A smart phone app
QNF_Friend. A family member, friend, or acquaintance
QNF_HealthPlan. Your health insurance plan
QNF_Staff. Someone at your doctor's office

Across:
1. Not at all
2. A little
3. A lot

BASE: if Q_Engage=1 ("No") show on same page as Qn_Facilitator

QNF_Other [O]
Please describe anything else that you think could help you **compare quality ratings** for health care services at different places (if you can't think of anything, just leave this blank):

I. DISCUSS

[Display]
For this next set of questions, **think only about your own individual health care** and not health care that was for someone else (e.g., a child, parent, or friend).

BASE: Total Qualified respondents

©2020 Kullgren JT et al. *JAMA Network Open*.

D_Engage [S]
In the past 12 months, did you **talk with a health care provider (e.g., a doctor, nurse, or pharmacist) about how much any health care services (e.g., a doctor visit, procedure, test, or medication) would cost you personally**?

1. Yes
2. No

BASE: D_Engage=1 ("yes")

DY_Services [GRID, S Across]
For each of the following types of health care services, did you **talk with a health care provider about the cost** in the past 12 months?

Down:
DY_Lab. A lab test (e.g., a blood or urine test)
DY_Image. An imaging or radiology test
DY_Meds. A prescription medicine
DY_Procedure. A procedure or surgery
DY_Outpatient. A visit to a doctor's office, a clinic, or some other place
DY_Inpatient. A hospitalization
DY_Other. Something else

Across:
1. Yes
2. No

BASE: DY_Other =1 ("yes") show on same page as dy_services

DY_Other_Specify [O]
For what other type(s) of health care services did you talk with a health care provider about the cost?

BASE: ask if D_Engage=1 ("yes")

DY_Effects/Facilitators [S]
Did **talking with a health care provider about the cost** for health care services in the past 12 months result in any of the following things for you?

Down:
DYE_GetCare. Getting a health care service you felt you needed.
DYE_PutOff. Deciding to put off getting a health care service until you could afford it.
DYE_Forego. Deciding a health care service wasn't worth the cost.
DYE_LessCost. Paying less for a health care service.
DYE_LessDebt. Having less debt from health care bills.
DYE_OtherCosts. Freeing up money you used to pay for other (non-health care) things.

Across:
1. Yes
2. No

BASE: ask if D_Engage=1 ("yes") show on same page as dy_effect

DYE_Other [O]

©2020 Kullgren JT et al. *JAMA Network Open*.

Please describe anything else that resulted from **talking with a health care provider about the cost** for health care services in the past 12 months (if nothing else resulted, just leave this blank):

BASE: ask if D_Engage=1 (“yes”)

[Grid, S Across]
How much did each of the following things help you **talk with a health care provider about the cost** for health care services in the past 12 months?

Down:
DYF_Web. A website
DYF_App. A smart phone app
DYF_Friend. A family member, friend, or acquaintance
DYF_HealthPlan. Your health insurance plan
DYF_Staff. Someone at your doctor’s office
DYF_Broker. An expert in money (e.g., financial adviser, broker, etc.)

Across:
1. Not at all
2. A little
3. A lot

BASE: ask if D_Engage=1 (“yes”) show on same page as on d_engage

DYF_Other[O]
Please describe anything else that helped you **talk with a health care provider about the cost** for health care services (if nothing else helped you, just leave this blank):

BASE: if DYF_Web=2 or 3 (“A little” or “A lot”)

Scripter: show DYF_Web_Specify - DYF_ Broker on same page

DYF_Web_Specify [O]
What website(s) helped you?

BASE: if DYF_App=2 or 3 (“A little” or “A lot”)

DYF_App_Specify [O]
What smart phone app(s) helped you?

BASE: if DYF_Staff=2 or 3 (“A little” or “A lot”)

DYF_Staff_Specify [O]
Who in your doctor’s office helped you?

BASE: if DYF_ Broker =2 or 3 (“A little” or “A lot”)

DYF_Broker_Specify [O]
What type of expert in money helped you?

BASE: if D_Engage=2 (“no”)

DN_Barriers/Facilitators[Grid,S across]

©2020 Kullgren JT et al. *JAMA Network Open*.

Were any of the following things reasons why you did not **talk with a health care provider about the cost** for health care services in the past 12 months?

Down:
DNB_NoNeed. You didn't need any health care services.
DNB_NotConsider. You didn't think about it.
DNB_TooHard. You felt it would be too difficult.
DNB_NotKnowHow. You didn't know how to.
DNB_NoImpact. It wouldn't change your decisions.
DNB_NoTime. You didn't have time to.
DNB_DocNoHelp. Providers can't do much about the cost of health care.

Across:
1. Yes
2. No

BASE: if D_Engage=2 ("no") show on same page dn_barriers

DNB_Other [O]
Please describe any other reasons you did not **talk with a health care provider about the cost** for health care services in the past 12 months (if there were no other reasons, just leave this blank):

BASE: if D_Engage=2 ("no")

[Grid,S across]

In the future, how much do you think each of the following things could help you **talk with a health care provider about the cost** for health care services?

Down:
DNF_Web. A website
DNF_App. A smart phone app
DNF_Friend. A family member, friend, or acquaintance
DNF_HealthPlan. Your health insurance plan
DNF_Staff. Someone at your doctor's office
DNF_Broker. An expert in money (e.g., financial adviser, broker, etc.)

Across:
1. Not at all
2. A little
3. A lot

BASE: if D_Engage=2 ("no") show on same page as dn_facilitator

DNF_Other [O]
Please describe anything else that you think could help you **talk with a health care provider about the cost** for health care services (if you can't think of anything, just leave this blank):

J. Negotiate

[Display]
For this next set of questions, **think only about your own individual health care** and not health care that was for someone else (e.g., a child, parent, or friend).

BASE: Total Qualified respondents

©2020 Kullgren JT et al. *JAMA Network Open*.

N_Engage [S]
In the past 12 months, did you **try to negotiate a lower price** for any health care services (e.g., a doctor visit, procedure, test, or medication), either before or after you received them?

1. Yes
2. No

BASE: N_Engage=1 ("yes")

NY_Services [S]
For each of the following types of health care services, did you **try to negotiate a lower price** in the past 12 months?

Down:
NY_Lab. A lab test (e.g., a blood or urine test)
NY_Image. An imaging or radiology test
NY_Meds. A prescription medicine
NY_Procedure. A procedure or surgery
NY_Outpatient. A visit to a doctor's office, a clinic, or some other place
NY_Inpatient. A hospitalization
NY_Other. Something else

Across:
1. Yes
2. No

BASE: NY_Other =1 ("yes") show on same page as Ny_services

NY_Other_Specify [O]
For what other types of health care services did you try to negotiate a lower price?

BASE: ask if N_Engage=1 ("yes")

NY_Effects/Facilitators [S]
Did **trying to negotiate a lower price** for health care services in the past 12 months result in any of the following things for you?

Down:
NYE_GetCare. Getting a health care service you felt you needed.
NYE_PutOff. Deciding to put off getting a health care service until you could afford it.
NYE_Forego. Deciding a health care service wasn't worth the cost.
NYE_LessCost. Paying less for a health care service.
NYE_LessDebt. Having less debt from health care bills.
NYE_OtherCosts. Freeing up money you used to pay for other (non-health care) things.
Across:
1. Yes
2. No

BASE: ask if N_Engage=1 ("yes") show on same page as NY_effects

NYE_Other [O]

Please describe anything else that resulted from **trying to negotiate a lower price** for health care services in the past 12 months (if nothing else resulted, just leave this blank):

©2020 Kullgren JT et al. *JAMA Network Open*.

BASE: ask if N_Engage=1 ("yes")

[Grid, S Across]
How much did each of the following things help you **try to negotiate a lower price** for health care services in the past 12 months?

Down:
NYF_Web. A website
NYF_App. A smart phone app
NYF_Friend. A family member, friend, or acquaintance
NYF_HealthPlan. Your health insurance plan
NYF_Staff. Someone at your doctor's office
NYF_Broker. An expert in money (e.g., financial adviser, broker, etc.)

Across:
1. Not at all
2. A little
3. A lot

BASE: ask if N_Engage=1 ("yes")

Scripter: show NYF_Other - NYF_Broker_Specify on same page

NYF_Other[O]
Please describe anything else that helped you **try to negotiate a lower price** for health care services in the past 12 months (if nothing else helped you, just leave this blank):

BASE: if NYF_Web=2 or 3 ("A little" or "a lot")

NYF_Web_Specify [O]
What website(s) helped you?

BASE: if NYF_App=2 or 3 ("A little" or "a lot")

NYF_App_Specify [O]
What smart phone app(s) helped you?

BASE: if NYF_Staff=2 or 3 ("A little" or "a lot")

NYF_Staff_Specify [O]
Who in your doctor's office helped you?

BASE: if NYF_ Broker =2 or 3 ("A little" or "a lot")

NYF_Broker_Specify [O]
What type of expert in money helped you?

BASE: if N_Engage=2 ("no")

NN_Barriers/Facilitators[Grid,S across]

Were any of the following things reasons why you did not **try to negotiate a lower price** for health care services in the past 12 months?

Down:

©2020 Kullgren JT et al. *JAMA Network Open*.

DNB_NoNeed. You didn't need any health care services.
DNB_NotConsider. You didn't think about it.
DNB_TooHard. You felt it would be too difficult.
DNB_NotKnowHow. You didn't know how to.
NNB_NoWorry. You were worried what others would think.
DNB_NoImpact. It wouldn't change your decisions.
DNB_NoTime. You didn't have time to.
DNB_CannotNeg. Patients can't negotiate prices for health care services.

Across:
1. Yes
2. No

BASE: if N_Engage=2 ("no") show on same page as NN_barriers page

NNB_Other [O]
Please describe any other reasons you did not **try to negotiate a lower price** for health care services in the past 12 months (if there were no other reasons, just leave this blank):

BASE: if N_Engage=2 ("no")

[Grid,S across]

In the future, how much do you think each of the following things could help you **try to negotiate a lower price** for health care services? Select one answer from each row in the grid.

Down:
NNF_Web. A website
NNF_App. A smart phone app
NNF_Friend. A family member, friend, or acquaintance
NNF_HealthPlan. Your health insurance plan
NNF_Staff. Someone at your doctor's office
NNF_Broker. An expert in money (e.g., financial adviser, broker, etc.)

Across:
1. Not at all
2. A little
3. A lot

BASE: if N_Engage=2 ("no") show on same page as NN_facilitators

NNF_Other [O]
Please describe anything else that you think could help you **try to negotiate a lower price** for health care services (if you can't think of anything, just leave this blank):

K. Other Behaviors

BASE: Total Qualified respondents

OtherBehavior [S]
In the past 12 months, **did you do anything else (that we did not ask about)** to try to make health care services more affordable for you?

1. Yes
2. No

©2020 Kullgren JT et al. *JAMA Network Open*.

BASE: Ask if OtherBehavior=1 ("yes")

OtherBehavior_Specify [O]
Please describe what you did and how much it helped you:

L. MRI

BASE: Total Qualified respondents
Scripter: Randomize Sections L. MRI and M. GASTROENTEROLOGIST
[The first section should see: In this next set of questions, we will ask you to imagine yourself in two different situations. Please read about the first situation carefully and then answer the questions that follow.**]**

[The Second section should see: In this next set of questions, we will ask you about the second situation. Please read carefully and then answer the questions that follow.]

[Display]

[Insert display text]

Imagine you hurt your lower back after lifting some heavy objects around the house. When your back pain didn't get better you went to see your primary care provider. Your primary care provider is now recommending you get **an MRI** (magnetic resonance imaging) of your lower back.

BASE: Total Qualified respondents

MRI_1 [Grid, S Across]
Please tell us **how much you disagree or agree** with the following statements. Select one answer from each row in the grid.

Comparing the price of getting the MRI at different places…

Down:
MRI_P_Difficult. …would be difficult.
MRI_P_Help. …would help me be able to get the MRI.
MRI_P_Save. …would help me pay less for the MRI.
MRI_P_Engage. …is something I would actually do.

Across:
1. Strongly Disagree
2. Disagree
3. Neutral
4. Agree
5. Strongly Agree

BASE: Total Qualified respondents

MRI_2 [Grid, S Across]
Please tell us **how much you disagree or agree** with the following statements. Select one answer from each row in the grid.

Talking with my primary care provider about the cost for the MRI…

Down:
MRI_D_Difficult. …would be difficult.
MRI_D_Help. …would help me be able to get the MRI.
MRI_D_Save. …would help me pay less for the MRI.

©2020 Kullgren JT et al. *JAMA Network Open*.

MRI_D_Engage. …is something I would actually do.

Across:
1. Strongly Disagree
2. Disagree
3. Neutral
4. Agree
5. Strongly Agree

BASE: Total Qualified respondents

MRI_3 [Grid, S Across]
Please tell us **how much you disagree or agree** with the following statements. Select one answer from each row in the grid.

Trying to negotiate a lower price for the MRI…

Down:
MRI_N_Difficult. …would be difficult.
MRI_N_Help. …would help me be able to get the MRI.
MRI_N_Save. …would help me pay less for the MRI.
MRI_N_Engage. …is something I would actually do.

Across:
1. Strongly Disagree
2. Disagree
3. Neutral
4. Agree
5. Strongly Agree

M. GASTROENTEROLOGIST

BASE: Total Qualified respondents

[Display]

[Insert display text]

Imagine you have been having stomach pain on and off for the last few months. When your stomach pain didn't get better you went to see your primary care provider. Your primary care provider is now recommending you see **a gastroenterologist** (a specialist who treats stomach problems).

Please tell us **how much you disagree or agree** with the following statements.

BASE: Total Qualified respondents

Gastro_1 [Grid, S Across]
Please tell us **how much you disagree or agree** with the following statements. Select one answer from each row in the grid.

Comparing the price of different gastroenterologists…

Down:
Spec_P_Difficult. …would be difficult.
Spec_P_Help. …would help me be able to see the gastroenterologist.
Spec_P_Save. …would help me pay less to see the gastroenterologist.
Spec_P_Engage. …is something I would actually do.

©2020 Kullgren JT et al. *JAMA Network Open*.

Across:
1. Strongly Disagree
2. Disagree
3. Neutral
4. Agree
5. Strongly Agree

BASE: Total Qualified respondents

Gastro_2 [Grid, S Across]

Comparing quality ratings for different gastroenterologists…

Down:
Spec_Q_Difficult. …would be difficult.
Spec_ Q _Help. …would help me be able to see the gastroenterologist.
Spec_ Q _Save. …would help me pay less to see the gastroenterologist.
Spec_Q _Engage. …is something I would actually do.

Across:
1. Strongly Disagree
2. Disagree
3. Neutral
4. Agree
5. Strongly Agree

BASE: Total Qualified respondents

Gastro_3 [Grid, S Across]
Please tell us **how much you disagree or agree** with the following statements. Select one answer from each row in the grid.

Talking with my primary care provider about the cost for seeing the gastroenterologist…

Down:
Spec_D_Difficult. …would be difficult.
Spec_D_Help. …would help me be able to see the gastroenterologist.
Spec_D_Save. …would help me pay less to see the gastroenterologist.
Spec_D_Engage. …is something I would actually do.

Across:
1. Strongly Disagree
2. Disagree
3. Neutral
4. Agree
5. Strongly Agree

BASE: Total Qualified respondents

Gastro_4 [Grid, S Across]
Please tell us **how much you disagree or agree** with the following statements. Select one answer from each row in the grid.

Trying to negotiate a lower price for seeing the gastroenterologist…

Down:

©2020 Kullgren JT et al. *JAMA Network Open*.

Spec_N_Difficult. …would be difficult.
Spec_N_Help. …would help me be able to see the gastroenterologist.
Spec_N_Save. …would help me pay less to see the gastroenterologist.
Spec_N_Engage. …is something I would actually do.

Across:
1. Strongly Disagree
2. Disagree
3. Neutral
4. Agree
5. Strongly Agree

N. Importance, Motivation, Control

[Display]

You are nearing the end of the survey! Thank you!

For this next set of questions, please rate your feelings on a scale from 1 to 10.

BASE: Total Qualified respondents

Import_Costs [Grid, S Across]
As of right now, **how important is it to you** to control how much you spend on health care services?

1	2	3	4	5	6	7	8	9	10

Not at all important **Extremely important**

BASE: Total Qualified respondents

Motiv_Costs [Grid, S Across]
As of right now, **how motivated are you** to control how much you spend on health care services?

1	2	3	4	5	6	7	8	9	10

Not at all motivated **Extremely motivated**

BASE: Total Qualified respondents

PC_Costs [Grid, S Across]
As of right now, **how much control do you feel you have** over how much you spend on health care services?

1	2	3	4	5	6	7	8	9	10

Not much control at all **A great deal of control**

BASE: Total Qualified respondents

Conf_Budget [Grid, S Across]
As of right now, **how confident are you that you could put aside enough money** to pay for health care services before you need them?

©2020 Kullgren JT et al. *JAMA Network Open*.

1	2	3	4	5	6	7	8	9	10

Not confident **Very confident**

BASE: Total Qualified respondents

Conf_Price [Grid, S Across]
As of right now, **how confident are you that you could compare prices** for health care services at different places?

1	2	3	4	5	6	7	8	9	10

Not confident **Very confident**

BASE: Total Qualified respondents

Conf_Quality [Grid, S Across]
As of right now, **how confident are you that you could compare quality ratings** for health care services at different places?

1	2	3	4	5	6	7	8	9	10

Not confident **Very confident**

BASE: Total Qualified respondents

Conf_Discuss [Grid, S Across]
As of right now, **how confident are you that you could talk with a health care provider (e.g., a doctor, nurse, or pharmacist) about the cost** for health care services?

1	2	3	4	5	6	7	8	9	10

Not confident **Very confident**

BASE: Total Qualified respondents

Conf_Negotiate [Grid, S Across]
As of right now, **how confident are you that you could try to negotiate lower prices** for health care services?

1	2	3	4	5	6	7	8	9	10

Very confident

Not confident

O. Financial Literacy

[Display]

The next few questions are about money.

BASE: Total Qualified respondents

©2020 Kullgren JT et al. *JAMA Network Open*.

Scripter : show FL_Interest - FL_Diversify on same page

FL_Interest [Grid, S Across]
Suppose you had $100 in a savings account and the interest rate was 2% per year. After 5 years, how much do you think you would have in the account if you left the money to grow?

1. More than $102
2. Exactly $102
3. Less than $102
4. Do not know

BASE: Total Qualified respondents

FL_ Inflation [Grid, S Across]
Imagine that the interest rate on your savings account was 1% per year and inflation was 2% per year. After 1 year, would you be able to buy with this account:

1. More than today
2. Exactly the same as today
3. Less than today
4. Do not know

BASE: Total Qualified respondents

FL_Diversify [Grid, S Across]
Do you think that the following statement is true or false? "Buying a single company stock usually provides a safer return than a stock mutual fund."

1. True
2. False
3. Do not know

P. Health Insurance Literacy

BASE: Total Qualified respondents

The final set of questions is about health insurance.

The first questions are about how confident you feel **choosing a health insurance plan**. Select one answer from each row in the grid.

How confident are you that…

Down:
HIL_Choosing_1. You understand health insurance terms?
HIL_Choosing_2. You know where to find the information you need to choose a health plan if you were not offered insurance through an employer?
HIL_Choosing_3. You know how to estimate what you have to pay for your health care needs in the next year, not including emergencies?
HIL_Choosing_4. You know where to go for help if you were having trouble affording health insurance outside an employer?
HIL_Choosing_5. You know what questions to ask so you can choose the best health plan for you?
HIL_Choosing_6. You would choose the health plan that is best for you?

Across:

©2020 Kullgren JT et al. *JAMA Network Open*.

1. Not at all confident
2. Slightly confident
3. Moderately confident
4. Very confident

BASE: Total Qualified respondents

The next questions are about **comparing health insurance plans**. When answering the questions, please imagine that you have a choice of health plans. Select one answer from each row in the grid.

When comparing health insurance plans, how likely are you to…

Down:
HIL_Comparing_1. Understand how the plans differ?
HIL_Comparing_2. Find out if you have to meet a deductible for health care services? A deductible is the amount of money you have to pay before your health insurance will pay anything for your health care.
HIL_Comparing_3. Look to see which doctors and hospitals are covered in each plan?
HIL_Comparing_4. Understand what you have to pay for prescription drugs?
HIL_Comparing_5. Understand what you would have to pay for emergency department visits?
HIL_Comparing_6. Understand what you would have to pay for specialist visits?
HIL_Comparing_7. Find out if the plans cover unexpected costs such as hospital stays?

Across:
1. Not at all likely
2. Somewhat likely
3. Moderately likely
4. Very likely

BASE: Total Qualified respondents

These questions ask how confident you feel about **using health insurance**. Select one answer from each row in the grid.

How confident are you that…

Down:
HIL_UsingC_1. You know how to find out what is and is not covered before you receive a health care service?
HIL_UsingC_2. You know how to figure out your share of the cost for care, after the health plan pays their share?
HIL_UsingC_3. You know what questions to ask your health plan if you have a coverage problem?
HIL_UsingC_4. You know what to do if your health plan refuses to pay for a service you think should be covered?

Across:
1. Not at all confident
2. Slightly confident
3. Moderately confident
4. Very confident

BASE: Total Qualified respondents

The last questions are about your experiences **using your health insurance plan**, for example, going to the doctor to receive care covered by the health plan. Select one answer from each row in the grid.

When using your health insurance plan, how likely are you to…

Down:
HIL_UsingL_1. Look into what your health plan will and will not cover before you get health care services?

©2020 Kullgren JT et al. *JAMA Network Open*.

HIL_UsingL_2. Look to member services to tell you what medical services your health plan covers?
HIL_UsingL_3. Find out if a doctor is in-network before you see him/her? A network is a group of doctors, hospitals, and other health care professionals who have a contract with a health insurance plan to take care of the plan's members.
HIL_UsingL_4. Review the statements you get from your health plan showing what you owe and what they paid for a service?

Across:
1. Not at all likely
2. Somewhat likely
3. Moderately likely
4. Very likely

BASE: Total Qualified respondents

[Display]

Thank you very much for completing our survey! Your answers will help us find new ways to help people who have to pay for much of their health care. If you have any questions, you can contact our Research Coordinator, [NAME], at [EMAIL ADDRESS].

[PRINCIPAL INVESTIGATOR NAME]
[PRINCIPAL INVESTIGATOR INSTITUTION]
[PRINCIPAL INVESTIGATOR EMAIL ADDRESS]

END OF QUESTIONNAIRE

©2020 Kullgren JT et al. *JAMA Network Open*.

eTable 1. Characteristics of Survey Respondents and Nonrespondents

Characteristic	Respondents (n=1637)	Non-Respondents (n=5023)	*P* value
	Percentage (95% CI)		
Male	53.9 (51.5 to 56.3)	43.5 (42.1 to 44.9)	< 0.001
Age in years[a]			
18 to 35	20.5 (18.5 to 22.4)	34.9 (33.5 to 36.2)	< 0.001
36 to 51	30.9 (28.7 to 33.1)	32.3 (31.0 to 33.6)	0.301
52 to 64	48.6 (46.2 to 51.0)	32.9 (31.6 to 34.2)	< 0.001
Race/Ethnicity			
White, non-Hispanic	81.6 (79.7 to 83.5)	69.0 (67.7 to 70.3)	< 0.001
Black, non-Hispanic	5.6 (4.5 to 6.7)	10.2 (9.3 to 11.0)	< 0.001
Hispanic	7.0 (5.7 to 8.2)	14.1 (13.2 to 15.1)	< 0.001
Other[b]	5.8 (4.7 to 6.9)	6.7 (6.0 to 7.4)	0.200
Education			
High school or less	19.4 (17.5 to 21.3)	21.3 (20.1 to 22.4)	0.111
Some college	26.4 (24.3 to 28.5)	32.8 (31.5 to 34.1)	< 0.001
Bachelor's degree	34.1 (31.9 to 36.4)	28.8 (27.6 to 30.1)	< 0.001
Master's degree or higher	20.0 (18.1 to 22.0)	17.1 (16.1 to 18.2)	0.007
Married	64.6 (62.3 to 66.9)	59.7 (58.3 to 61.0)	< 0.001
Employed	83.3 (81.5 to 85.1)	81.1 (80.0 to 82.1)	0.046
Household income[c]			
< 250 % FPL	20.8 (18.9 to 22.8)	28.8 (27.5 to 30.0)	< 0.001
250 to 400 % FPL	25.5 (23.4 to 27.6)	25.1 (23.9 to 26.3)	0.751
400+ % FPL	53.7 (51.3 to 56.1)	46.1 (44.7 to 47.5)	< 0.001
US Census region			
Northeast	15.9 (14.2 to 17.7)	17.6 (16.6 to 18.7)	0.116
Midwest	33.6 (31.3 to 35.9)	25.8 (24.6 to 27.1)	< 0.001
South	31.6 (29.3 to 33.8)	33.7 (32.4 to 35.0)	0.113
West	18.9 (17.0 to 20.8)	22.8 (21.6 to 24.0)	0.001
Fair or poor health	10.0 (8.5 to 11.4)	10.8 (9.9 to 11.6)	0.363
Source of health insurance			
Employer	81.7 (79.8 to 83.5)	86.3 (85.3 to 87.3)	< 0.001
Marketplace	11.5 (10.0 to 13.1)	8.1 (7.3 to 8.8)	< 0.001
Other	6.8 (5.6 to 8.0)	5.6 (5.0 to 6.3)	0.083
Out-of-pocket costs in last 12 months			
$0	26.1 (24.0 to 28.3)	36.1 (34.8 to 37.4)	< 0.001
$1 to $999	36.2 (33.9 to 38.6)	38.6 (37.3 to 40.0)	0.078
$1,000 or more	37.6 (35.3 to 40.0)	25.2 (24.0 to 26.4)	< 0.001
Satisfied with insurance	63.6 (61.3 to 65.9)	71.2 (70.0 to 72.5)	< 0.001

FPL = Federal Poverty Level; CI = Confidence Interval

[a]Ages grouped into generational categories (Millennials, Generation X, and Baby Boomers respectively) as defined by the Pew Research Center.

[b]"Other" includes American Indian or Alaska Native, Asian, Native Hawaiian/Pacific Islander, and those who selected more than one racial category.

[c]Annual household income is captured in 19 different categories ranging from "Less than $5,000" to "$175,000 or more."

©2020 Kullgren JT et al. *JAMA Network Open*.

eTable 2. Characteristics of Survey Sample and Nonelderly 2015 NHIS Participants in HDHPs			
**Characteristic**	**Weighted percentage (95% CI) from survey sample (n = 1637)**	**Weighted percentage (95% CI) from 2015 NHIS (n = 4701)**	***P* value**
Female	50.6 (47.7 to 53.6)	50.9 (49.0 to 52.7)	0.887
Age[a]			
18 to 35	27.5 (24.6 to 30.4)	33.2 (31.3 to 35.2)	0.001
36 to 51	35.7 (32.8 to 38.6)	36.3 (34.5 to 38.1)	0.710
52 to 64	36.8 (34.1 to 39.5)	30.4 (28.6 to 32.3)	< 0.001
Race/ethnicity			
White	75.4 (72.5 to 78.3)	77.2 (75.5 to 78.8)	0.286
Black	6.5 (4.8 to 8.1)	6.5 (5.7 to 7.4)	0.987
Hispanic	9.5 (7.5 to 11.4)	8.5 (7.4 to 9.5)	0.368
Other[b]	8.6 (6.5 to 10.7)	5.8 (4.9 to 6.7)	0.013
Education			
Less than high school	3.0 (1.6 to 4.5)	4.7 (3.9 to 5.5)	0.042
High school	20.9 (18.4 to 23.4)	17.5 (15.9 to 19.1)	0.024
Some college or more	76.0 (73.4 to 78.7)	77.7 (76.0 to 79.5)	0.298
Married	65.6 (62.7 to 68.4)	63.8 (61.9 to 65.7)	0.299
Children in household	36.0 (33.0 to 38.9)	42.4 (40.0 to 44.7)	0.001
Working	83.8 (81.6 to 86.0)	84.2 (82.8 to 85.6)	0.761
Household income			
< 100 % FPL	2.2 (1.4 to 3.1)	2.5 (1.9 to 3.1)	0.629
100 to 199 % FPL	7.1 (5.8 to 8.4)	9.0 (7.9 to 10.1)	0.031
200 to 399 % FPL	31.9 (29.2 to 34.6)	30.1 (28.3 to 32.0)	0.298
400+ % FPL	58.8 (55.9 to 61.7)	58.4 (56.4 to 60.4)	0.815
US Census region			
Northeast	17.9 (15.5 to 20.3)	14.2(12.5 to 15.9)	0.014
Midwest	27.6 (25.1 to 30.1)	28.5(26.6 to 30.5)	0.568
South	34.8(31.9 to 37.7)	36.2 (33.9 to 38.5)	0.456
West	19.7 (17.3 to 22.1)	21.1 (19.3 to 22.8)	0.367
Fair or poor health status	9.4 (7.7 to 11.2)	4.8 (4.0 to 5.6)	< 0.001
Insurance from employer	84.6 (82.7 to 86.6)	84.9 (83.7 to 86.1)	0.833
Chronic condition[c]	31.1 (28.5 to 33.7)	31.1 (29.2 to 33.0)	0.990

HDHPs = high-deductible health plans; NHIS = National Health Interview Survey; FPL = Federal Poverty Level
[a]Ages grouped into generational categories (Millennials, Generation X, and Baby Boomers respectively) as defined by the Pew Research Center.
[b]"Other" includes American Indian or Alaska Native, Asian, Native Hawaiian/Pacific Islander, and those who selected more than one racial category.
[c]Asthma, chronic bronchitis, chronic obstructive pulmonary disease, diabetes, coronary heart disease, or high blood pressure. This measure of chronic conditions differs from the measure used in the main GfK survey because the 2015 NHIS did not ask participants about diagnoses of an anxiety disorder or depression.

©2020 Kullgren JT et al. *JAMA Network Open*.

eTable 3. Characteristics of Americans in High-Deductible Health Plans (n = 1637)	
**Characteristic**	**% (95% CI) [a]**
Age in years[b]	
18 to 35	27.5 (24.6 to 30.4)
36 to 51	35.7 (32.8 to 38.6)
52 to 64	36.8 (34.1 to 39.5)
Female	50.6 (47.7 to 53.6)
Race/ethnicity	
White, non-Hispanic	75.4 (72.5 to 78.3)
Black, non-Hispanic	6.5 (4.9 to 8.1)
Hispanic	9.5 (7.5 to 11.4)
Other[c]	8.6 (6.5 to 10.7)
Education	
High school or less	24.0 (21.3 to 26.6)
Some college	29.4 (26.7 to 32.2)
College degree	28.6 (26.1 to 31.1)
Master's degree or higher	18.0 (15.9 to 20.1)
Household income[d]	
< 250 % FPL	15.9 (14.0 to 17.6)
250 to 400 % FPL	25.3 (22.7 to 27.8)
400 + % FPL	58.8 (55.9 to 61.7)
Employed	83.8 (81.6 to 86.0)
US Census region	
Northeast	17.9 (15.5 to 20.3)
Midwest	27.6 (25.2 to 30.1)
South	34.8 (31.9 to 37.7)
West	19.7 (17.3 to 22.1)
Fair or poor health	9.3 (7.6 to 11.1)
Chronic condition[e]	42.4 (39.6 to 45.3)
Source of health insurance	
Employer	84.6 (82.6 to 86.5)
Marketplace	9.1 (7.7 to 10.8)
No health savings account[f]	32.5 (29.8 to 35.3)

FPL = Federal Poverty Level
[a]Weighted estimates of the percentage of individuals with each characteristic.
[b]Generational categories (Millennials, Generation X, and Baby Boomers respectively) as defined by the Pew Research Center.
[c]American Indian or Alaska Native, Asian, Native Hawaiian/Pacific Islander, or more than one racial category.
[d]Annual household income measured in 19 different categories ranging from "less than $5,000" to "$175,000 or more."
[e]Anxiety disorder, asthma, bronchitis, chronic obstructive pulmonary disease, depression, type 1 or type 2 diabetes, heart attack, heart disease, or high blood pressure.
[f]Health savings account defined as in the National Health Interview Survey as "a special account or fund that can be used to pay for medical expenses" that are "sometimes referred to as Health Savings Accounts (HSAs), Health Reimbursement

©2020 Kullgren JT et al. *JAMA Network Open*.

Accounts (HRAs), Personal Care accounts, Personal Medical funds, or Choice funds, and are different from Flexible Spending Accounts."

©2020 Kullgren JT et al. *JAMA Network Open*.

eTable 4. Coefficients From Logit Regression Model to Predict Not Having an HSA (n = 1547)[a]		
**Characteristic**	**Coefficient (95% CI)**	***P* value**
Age group (reference 18 to 35)[b]		
36 to 51	-0.31 (-0.72 to 0.10)	0.14
51 to 64	-0.09 (-0.50 to 0.33)	0.69
Male	0.04 (-0.27 to 0.35)	0.79
Race/ethnicity (reference White, non-Hispanic)		
Black	-0.10 (-0.84 to 0.64)	0.79
Hispanic	0.005 (-0.55 to 0.54)	0.98
Other[c]	0.17 (-0.42 to 0.77)	0.57
Education (reference high school or less)		
Some college	-0.33 (-0.77 to 0.11)	0.15
Bachelor's degree	-0.43 (-0.90 to 0.03)	0.07
Master's degree or higher	-1.02 (-1.56 to -0.48)	< 0.001
Income (reference less than 250% FPL)		
250 to 400% FPL	-0.45 (-0.87 to -0.04)	0.03
≥ 400% FPL	-0.26 (-0.66 to 0.14)	0.21
Region of residence (reference Northeast)		
Midwest	-0.21 (-0.65 to 0.23)	0.34
South	0.36 (-0.08 to 0.80)	0.11
West	0.40 (-0.11 to 0.91)	0.13
Chronic condition	-0.11 (-0.43 to 0.20)	0.48
Consumer engagement score (reference lowest tertile)[d]		
Middle tertile	0.28 (-0.09 to 0.66)	0.14
Highest tertile	0.24 (-0.15 to 0.63)	0.22
Financial literacy score (reference lowest tertile)[e]		
Middle tertile	0.18 (-0.25 to 0.60)	0.41
Highest tertile	0.07 (-0.34 to 0.48)	0.75
Health insurance literacy score (reference lowest tertile)[f]		
Middle tertile	0.20 (-0.15 to 0.55)	0.26
Highest tertile	-0.08 (-0.47 to 0.30)	0.67
Health status (reference excellent)		
Very Good	0.22 (-0.27 to 0.71)	0.38
Good	0.77 (0.25 to 1.30)	0.004
Fair	0.75 (0.08 to 1.43)	0.03
Poor	0.41 (-0.66 to 1.48)	0.45
Insurance source (reference employer without plan choice)		
Employer with choice of plans	-0.80 (-1.12 to -0.46)	< 0.001
Insurance exchange[g]	1.52 (1.03 to 2.01)	< 0.001
Other source	1.16 (0.63 to 1.70)	< 0.001
HSA = Health Savings Account; CI = confidence interval; FPL = Federal Poverty Level.		

©2020 Kullgren JT et al. *JAMA Network Open*.

[a]HSA defined as in the National Health Interview Survey as "a special account or fund that can be used to pay for medical expenses" that are "sometimes referred to as Health Savings Accounts (HSAs), Health Reimbursement Accounts (HRAs), Personal Care accounts, Personal Medical funds, or Choice funds, and are different from Flexible Spending Accounts." Sample is the 1,547 respondents with non-missing covariates.
[b]Ages grouped to represent distinct generations (Millennials, Generation X, and Baby Boomers respectively) according to Pew Research Center groupings.
[c]"Other" includes American Indian or Alaska Native, Asian, Native Hawaiian/Pacific Islander, and those who selected more than one racial category.
[d]Tertiles of participants' scores for the Altarum Consumer Engagement Measure™ which is a scale of questions representing three distinct subscales of patient engagement with their health and healthcare (informed choice, navigation, and ownership).
[e]Tertiles of participants' sum of scores for 3 measures of financial literacy developed by Lusardi and Mitchell.
[f]Tertiles of participants' scores for the Health Insurance Literacy Measure.
[g]Respondents answered they had "health insurance that you bought through a state or federal individual marketplace/exchange."

©2020 Kullgren JT et al. *JAMA Network Open*.

eTable 5. Coefficients From Logit Regression Model to Predict Not Saving in an HSA in the Last 12 Months (n = 864)[a]		
**Characteristic**	**Coefficient (95% CI)**	***P* value**
Age group (reference 18 to 35)[b]		
36 to 51	-0.34 (-0.80 to 0.11)	0.14
51 to 64	-0.27 (-0.74 to 0.21)	0.27
Male	0.32 (-0.04 to 0.68)	0.08
Race/ethnicity (reference White, non-Hispanic)		
Black	0.64 (-0.13 to 1.41)	0.10
Hispanic	-0.32 (-0.97 to 0.32)	0.33
Other[c]	0.65 (-0.19 to 1.49)	0.13
Education (reference high school or less)		
Some college	-0.16 (-0.72 to 0.41)	0.59
Bachelor’s degree	-0.57 (-1.13 to -0.02)	0.04
Master’s degree or higher	-0.72 (-1.31 to -0.13)	0.02
Income (reference less than 250% FPL)		
250 to 400% FPL	-0.03 (-0.62 to 0.56)	0.92
≥ 400% FPL	-0.24 (-0.76 to 0.30)	0.39
Region of residence (reference Northeast)		
Midwest	-0.48 (-0.97 to 0.01)	0.05
South	-0.28 (-0.80 to 0.25)	0.30
West	-0.19 (-0.77 to 0.39)	0.52
Chronic condition	-0.05 (-0.42 to 0.33)	0.81
Consumer engagement score (reference lowest tertile)[d]		
Middle tertile	0.31 (-0.12 to 0.75)	0.16
Highest tertile	0.16 (-0.26 to 0.59)	0.45
Financial literacy score (reference lowest tertile)[e]		
Middle tertile	-0.03 (-0.58 to 0.53)	0.92
Highest tertile	-0.31 (-0.84 to 0.23)	0.26
Health insurance literacy score (reference lowest tertile)[f]		
Middle tertile	-0.08 (-0.50 to 0.35)	0.73
Highest tertile	-0.49 (-0.92 to -0.05)	0.03
Health status (reference excellent)		
Very Good	-0.12 (-0.64 to 0.40)	0.65
Good	-0.28 (-0.84 to 0.28)	0.33
Fair	-0.24 (-1.10 to 0.61)	0.58
Poor	0.88 (-1.45 to 3.22)	0.56
Insurance source (reference employer without plan choice)		
Employer with choice of plans	-0.39 (-0.79 to 0.02)	0.06
Insurance exchange[g]	-1.35 (-2.61 to -0.10)	0.03
Other source	-0.37 (-1.25 to 0.51)	0.41
HSA = Health Savings Account; CI = confidence interval; FPL = Federal Poverty Level.		

©2020 Kullgren JT et al. *JAMA Network Open*.

[a]HSA defined as in the National Health Interview Survey as "a special account or fund that can be used to pay for medical expenses" that are "sometimes referred to as Health Savings Accounts (HSAs), Health Reimbursement Accounts (HRAs), Personal Care accounts, Personal Medical funds, or Choice funds, and are different from Flexible Spending Accounts." HSA savings (the dependent variable) was defined as the level of money saved in the last 12 months if a respondent reported having an HSA and saving money for health care through an HSA. Categories of savings levels were $0 (defined as either reporting not saving any money for health care or not saving any money for health care through their HSA), less than $500, $500 to $999, $1,000 to $1,499, $1,500 to $1,999, or $2,000 or more. Sample was anyone who reported having an HSA and had non-missing values for covariates in model (n = 864). Survey weights were based on the full sample of respondents with non-missing covariates (n = 1,564).
[b]Ages grouped to represent distinct generations (Millennials, Generation X, and Baby Boomers respectively) according to Pew Research Center groupings.
[c]"Other" includes American Indian or Alaska Native, Asian, Native Hawaiian/Pacific Islander, and those who selected more than one racial category.
[d]Tertiles of participants' scores for the Altarum Consumer Engagement Measure™ which is a scale of questions representing three distinct subscales of patient engagement with their health and healthcare (informed choice, navigation, and ownership).
[e]Tertiles of participants' sum of scores for 3 measures of financial literacy developed by Lusardi and Mitchell.
[f]Tertiles of participants' scores for the Health Insurance Literacy Measure.
[g]Respondents answered they had "health insurance that you bought through a state or federal individual marketplace/exchange."

©2020 Kullgren JT et al. *JAMA Network Open*.
